# SCF^β-TRCP^ promotes cell growth by targeting PR-Set7/Set8 for degradation

**DOI:** 10.1038/ncomms10185

**Published:** 2015-12-15

**Authors:** Zhiwei Wang, Xiangpeng Dai, Jiateng Zhong, Hiroyuki Inuzuka, Lixin Wan, Xiaoning Li, Lixia Wang, Xiantao Ye, Liankun Sun, Daming Gao, Lee Zou, Wenyi Wei

**Affiliations:** 1 The Cyrus Tang Hematology Center and Collaborative Innovation Center of Hematology, Jiangsu Institute of Hematology, the First Affiliated Hospital, Soochow University, Suzhou 215123, P.R. China; 2 Department of Pathology, Beth Israel Deaconess Medical Center, Harvard Medical School, Boston, Massachusetts 02215, USA; 3 Department of Pathology, Xinxiang Medical University, Xinxiang 453000, P.R. China; 4 Department of Pathophysiology, Basic Medical College, Jilin University, Changchun 130021, P.R. China; 5 State Key Laboratory of Cell Biology, Institute of Biochemistry and Cell Biology, Shanghai Institutes for Biological Sciences, Chinese Academy of Sciences, 320 Yue-yang Road, Shanghai 200031, P.R. China; 6 Department of Pathology, Massachusetts General Hospital Cancer Center, Harvard Medical School, Boston, Massachusetts 02129, USA

## Abstract

The Set8/PR-Set7/KMT5a methyltransferase plays critical roles in governing transcriptional regulation, cell cycle progression and tumorigenesis. Although CRL4^Cdt2^ was reported to regulate Set8 stability, deleting the PIP motif only led to partial resistance to ultraviolet-induced degradation of Set8, indicating the existence of additional E3 ligase(s) controlling Set8 stability. Furthermore, it remains largely undefined how DNA damage-induced kinase cascades trigger the timely destruction of Set8 to govern tumorigenesis. Here, we report that SCF^β-TRCP^ earmarks Set8 for ubiquitination and degradation in a casein kinase I-dependent manner, which is activated by DNA-damaging agents. Biologically, both CRL4^Cdt2^ and SCF^β-TRCP^-mediated pathways contribute to ultraviolet-induced Set8 degradation to control cell cycle progression, governing the onset of DNA damage-induced checkpoints. Therefore, like many critical cell cycle regulators including p21 and Cdt1, we uncover a tight regulatory network to accurately control Set8 abundance. Our studies further suggest that aberrancies in this delicate degradation pathway might contribute to aberrant elevation of Set8 in human tumours.

## Abstract (web summary)

The Set8-Set7 methyltransferase plays a critical role in cell cycle progression and tumorigenesis, and is degraded through modification by the E3 ubiquitin ligase CRL4^Cdt2^. Here Wang *et al.* identify SCF^β-TRCP^ as an additional E3 ubiquitin ligase that marks Set8 for degradation in response to DNA damage.

## Graphical abstract

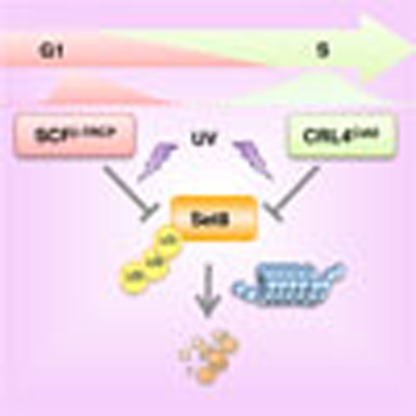

Post-translational modifications of histones play a critical role in a number of cellular processes such as mitosis, meiosis and the DNA damage response^1^. These modifications include methylation, acetylation, phosphorylation and ubiquitination, which often occur on the N-terminal tails of histone proteins. Importantly, emerging evidence has suggested that histone methylation is one of the important post-translational modifications with pivotal biological consequences. Specifically, it has been revealed that histone H4 Lys 20 (H4K20) is one of the methylated lysine residues on the H4 N-terminal tail. In mammals, three methyltransferases including Set8, Suv4-20h1 and Suv4-20h2 have been identified to regulate the H4K20 methylation (mono-, di- and tri-methylation) status^1^. Among these methyltransferases, Suv4-20h1/h2 promotes the transition from H4K20me1 to di- and tri-methylation of H4K20 (H4K20me2/3)^2^. On the other hand, the Set8/PR-Set7/lysine methyltransferase 5a (KMT5a), a sole monomethyltransferase, primarily regulates the monomethylation of H4K20 (refs 3, 4). Knockout mouse studies have further revealed that Set8 is required for developmental processes and loss of Set8 could cause cell cycle defects and increased DNA damage^3^,^4^. Besides H4K20, Set8 has also been found to methylate non-histone proteins including the p53 tumour-suppressor protein, subsequently preventing p53 from promoter binding to inhibit the transcriptional activation of p21 and p53 upregulated modulator of apoptosis (PUMA)^5^.

Several lines of evidence have defined that Set8 exerts its biological functions in regulating cell cycle and DNA damage response in part through its interaction with a number of nuclear proteins, such as proliferating cell nuclear antigen (PCNA), RNA polymerase II, ER, LEF3 and TWIST^1^. As Set8 plays an important role in various cellular processes, its activity needs to be tightly regulated for the precise control of cell fate. To this end, a wealth of evidence has revealed that Set8 could be regulated at both the transcriptional level^6^ and by post-translational modification(s)^1^. Multiple enzymes such as kinases, small ubiquitin-like modifier, and ubiquitin ligases have been reported to control Set8 modification. For example, Cyclin B/Cdk1 phosphorylates Set8 at Ser29 during mitosis^7^, and the E3 ubiquitin ligase complex, CRL4^Cdt2^, governs ubiquitination-mediated Set8 degradation^4^,^8^,^9^. In addition, the E3 ubiquitin ligases SCF^Skp2^ and B-lymphoma and BAL-associated protein have also been reported to regulate Set8 stability^10^,^11^, but no physiological evidence has been obtained to support whether B-lymphoma and BAL-associated protein or SCF^Skp2^ directly ubiquitinates Set8. Moreover, the anaphase-promoting complex APC^Cdh1^ has also recently been found to promote the ubiquitination of Set8 and subsequent proteolysis^7^. However, although Set8 destruction has been reported to be stimulated by ultraviolet^12^, it remains largely unclear whether DNA damage-induced kinase cascades play a critical role in this process. In the present study, we report that Set8 is an ubiquitin substrate of β-TRCP (β-transducin repeat-containing protein), and Set8 ubiquitination and subsequent degradation is timely governed by the E3 ubiquitin ligase SCF^β-TRCP^ in a casein kinase I (CKI)-dependent manner.

β-TRCP is one of the 69 F-box proteins that form the SCF (Skp1-Cullin-1-F-box protein) type of E3 ligase complexes. Notably, the SCF complex is composed of Skp1, Cullin-1, Ring box protein-1 (Rbx1)/Roc1 and one of the 69 F-box proteins. SCF^β-TRCP^ often targets substrates containing the consensus sequence DSGXXS degron^13^. Moreover, SCF^β-TRCP^-mediated ubiquitination and degradation requires specific kinases to phosphorylate two serine residues within the phosphodegron of its substrates^13^. A growing list of SCF^β-TRCP^ ubiquitin substrates have recently been identified including EMI-1 (early mitotic inhibitor-1)^14^,^15^, Wee1 (ref. 16), and Cdc25A (cell division cycle 25 homologue A)^17^,^18^. Biologically, these substrates regulate cell cycle and cellular apoptosis, indicating that β-TRCP is critically involved in governing proper cell cycle progression and cell survival^13^. Here, we report that Set8 interacts with the SCF^β-TRCP^ complex and depletion of endogenous β-TRCP leads to an accumulation of the Set8 protein. Moreover, our results reveal a critical role of the CKI kinase in SCF^β-TRCP^-mediated degradation of Set8. Furthermore, inhibition of CKI-mediated phosphorylation of Set8 at Ser253 suppresses its destruction by SCF^β-TRCP^. More importantly, β-TRCP-mediated degradation of Set8 affects cell growth and cell cycle progression. Hence, our current study supports a pivotal role of β-TRCP in CKI-mediated Set8 degradation, and further implies that targeting β-TRCP could be a novel approach to govern cell cycle progression in part by regulating Set8 destruction.

## Results

### Set8 interacts with SCF^β-TRCP^ E3 ubiquitin ligase complex

**Figure 1:**
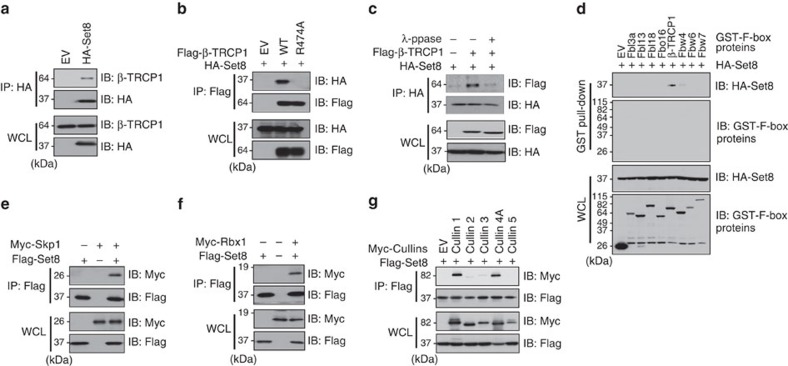
Set8 interacts with the SCF^β-TRCP^ complex. (**a**) Immunoblot (IB) analysis of whole cell lysates (WCL) and immunoprecipitates (IP) derived from HeLa cells transfected with HA-Set8 or empty vector (EV) as a negative control. (**b**) IB analysis of WCL and IP derived from HeLa cells transfected with HA-Set8 and Flag-tagged wild type or R474A mutant β-TRCP1 constructs, or EV as a negative control. (**c**) IB analysis of WCL and IP derived from HeLa cells transfected with HA-Set8 and Flag-β-TRCP1 constructs as indicated. Where indicated, cell lysates were pre-treated with λ-phosphatase before the IP procedure. (**d**) IB analysis of WCL and GST pull-down derived from HeLa cells transfected with HA-Set8 and the indicated GST-F-box proteins. (**e**) IB analysis of WCL and IP derived from HeLa cells transfected with Flag-Set8 and Myc-Skp1 constructs, as indicated. (**f**) IB analysis of WCL and IP derived from HeLa cells transfected with Flag-Set8 and Myc-Rbx1 constructs, as indicated. (**g**) IB analysis of WCL and IP derived from HeLa cells transfected with Flag-Set8 and Myc-Cullin1, 2, 3, 4A or 5 expression plasmids.

To better understand the critical role of the Set8 methyltransferase in carcinogenesis, we employed an affinity purification coupled with the mass spectrometry approach to identify proteins associated with Set8 (refs 19, 20, 21). Our mass spectrometry results showed that Set8 co-purified with multiple proteins including PCNA^4^,^22^ and β-TRCP (Supplementary Fig. 1a). To validate β-TRCP as a novel Set8-interacting protein, we performed co-immunoprecipitation experiments and observed that exogenously expressed Set8 associated with endogenous β-TRCP1 (Fig. 1a and Supplementary Fig. 1b,c). We further demonstrated that the interaction of β-TRCP1 and Set8 was significantly reduced using a β-TRCP1 mutant (R474A) harbouring a point mutation within the substrate interaction site of β-TRCP1 (ref. 13; Fig. 1b and Supplementary Fig. 1d). It is well characterized that proper phosphorylation of substrates by kinases is required for SCF^β-TRCP^-mediated ubiquitination and degradation^13^,^23^. In keeping with this notion, we found that the β-TRCP/Set8 interaction was largely abolished by the phosphatase treatment (Fig. 1c). More importantly, using a panel of F-box proteins, we observed that Set8 specifically bound β-TRCP1, but not other F-box proteins we examined (Fig. 1d), further suggesting for a specific interaction between Set8 and β-TRCP1. As additional supporting evidence, we identified Set8 to interact with numerous known SCF components including Skp1 (Fig. 1e) and Rbx1 (Fig. 1f), indicating that the SCF^β-TRCP^ holo-complex may be involved in governing Set8 stability. Moreover, in addition to the reported interaction between Set8 and Cullin 4A (refs 4, 8, 9), we observed that Set8 also interacted with Cullin 1, but not other Cullin family members in cells (Fig. 1g and Supplementary Fig. 1e).

### Set8 stability is negatively controlled by β-TRCP

**Figure 2:**
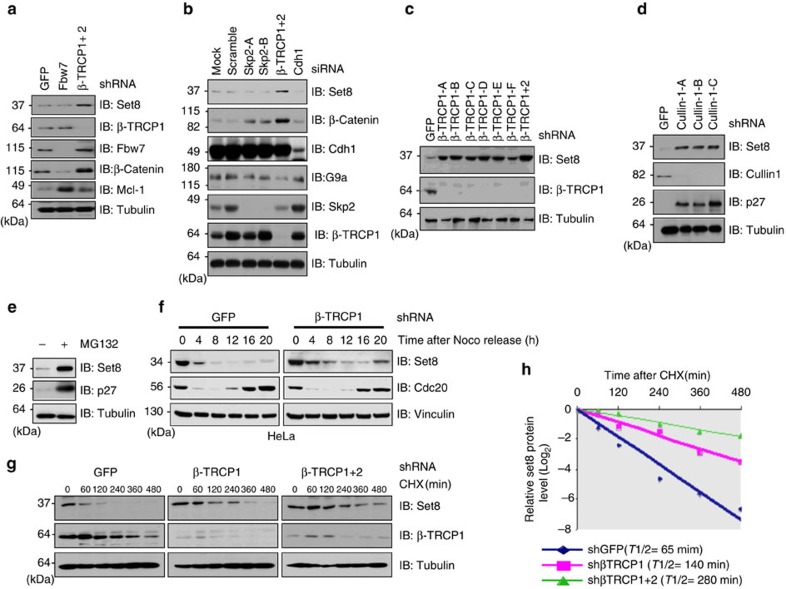
Set8 protein stability is negatively controlled by the SCF^β-TRCP1^ E3 ligase complex. (**a**) Immunoblot (IB) analysis of whole cell lysates (WCL) derived from HeLa cells infected with the indicated shRNA constructs. (**b**) IB analysis of HeLa cells transfected with the indicated siRNA oligonucleotides. (**c**) IB analysis of WCL derived from HeLa cells infected with shRNA constructs specific for GFP, β-TRCP1 (six independent lentiviral β-TRCP1-targeting shRNA constructs namely, -A, -B, -C, -D, -E, -F), or β-TRCP1+2, followed by selection with 1 μg ml^−1^ puromycin for 3 days to eliminate non-infected cells. (**d**) IB analysis of WCL from 293 T cells infected with shRNA specific for GFP, or several shRNA constructs against Cullin 1 (three independent lentiviral Cullin 1-targeting shRNA constructs namely, -A, -B, -C) followed by selection with 1 μg ml^−1^ puromycin for 3 days to eliminate the non-infected cells. (**e**) IB analysis of WCL derived from 293 T cells with or without MG132 treatment. (**f**) HeLa cells were infected with the shGFP or shβ-TRCP1 followed by selection with 1 μg ml^−1^ puromycin for 3 days to eliminate non-infected cells. The generated stable cell lines were then treated with nocodazole to arrest at the M phase, and then release back to the cell cycle by washing off nocodazole. At the indicated time points, WCL were prepared and immunoblots were probed with the indicated antibodies. (**g**) HeLa cells were infected with the shRNA constructs for GFP, β-TRCP1 or β-TRCP1+2 followed by selection with 1 μg ml^−1^ puromycin for 3 days to eliminate non-infected cells. The generated stable cell lines were then split into 60-mm dishes. After 20 h, cells were treated with 20 μg ml^−1^ cycloheximide (CHX). At the indicated time points, WCL were prepared and immunoblots were probed with the indicated antibodies. (**h**) Quantification of the Set8 band intensities in **g**. Set8 band intensity was normalized to tubulin, and then normalized to the *t*=0 controls.

To determine whether Set8 is a *bone fide* substrate of SCF^β-TRCP^, we next assessed Set8 protein abundance changes in cells after depleting endogenous β*-TRCP*. Notably, we found that depletion of endogenous β*-TRCP1*, but not other F-box proteins we examined, led to an elevation in Set8 protein levels (Fig. 2a–c and Supplementary Fig. 2a–c). In keeping with the notion that both Cullin1 and Cullin 4 were involved in regulation of Set8 stability, an increase in Set8 abundance was observed after depleting either endogenous *Cullin 1* or endogenous *Cullin 4A*, but not *Cullin 4B* (Fig. 2d and Supplementary Fig. 2d,e). However, we did not observe an additive elevation in the abundance of endogenous Set8 protein in *Cullin 4A* and *Cullin 4B* double knockdown cells compared with either protein depleted individually (Supplementary Fig. 2e), indicating that Cullin 4A, but not Cullin 4B plays a critically important physiological role in the regulation of Set8 degradation in cells. In further support of this notion, we observed that Culllin 4A, but not Cullin 4B interacts with Set8 in cells (Supplementary Fig. 2f,g). As such, there are elevated Set8 protein levels upon genetic deletion of *Culin 4A*, but not *Cullin 4B* in mouse embryonic fibroblasts (Supplementary Fig. 2h,i).

Moreover, treatment with the 26S proteasome inhibitor MG132 caused an upregulation of Set8 protein levels, indicating the involvement of the 26S proteasome in governing Set8 stability (Fig. 2e and Supplementary Fig. 2j). Furthermore, compared with control cells, depletion of endogenous β*-TRCP1* led to an elevation of Set8 protein abundance in the G1 phase (Fig. 2f and Supplementary Fig. 2k), largely through extending the half-life of endogenous Set8 protein (Fig. 2g,h and Supplementary Fig. 2l), indicating that β-TRCP primarily governs Set8 abundance through a post-transcriptional mechanism.

### CKI is involved in the regulation of Set8 stability

**Figure 3:**
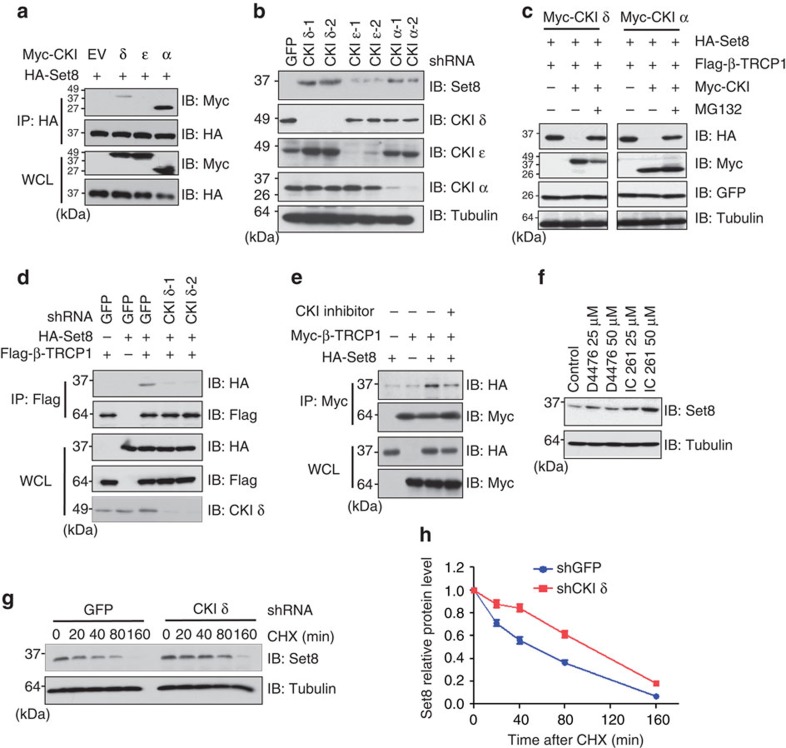
Casein Kinase Iδ (CKIδ) controls Set8 stability. (**a**) Immunoblot (IB) analysis of whole cell lysates (WCL) and immunoprecipitates (IP) derived from HeLa cells transfected with HA-Set8 and Myc-tagged versions of the indicated CKI isoforms. (**b**) IB analysis of HeLa cells that were infected with shRNA specific for GFP or the indicated CKI isoforms (two independent shRNAs, namely -1 and -2), followed by selection with 1 μg ml^−1^ puromycin for 3 days to eliminate non-infected cells. (**c**) IB analysis of WCL derived from HeLa cells transfected with HA-Set8, Flag-β-TRCP and indicated kinases. Where indicated, cells were treated with MG132. (**d**) IB analysis of WCL and IP derived from HeLa cells infected with shGFP or shCKIδ followed by selection with 1 μg ml^−1^ puromycin for 3 days to eliminate non-infected cells. (**e**) IB analysis of WCL and IP derived from HeLa cells transfected with HA-Set8 and Myc-β-TRCP1. Where indicated, cells were treated with the CKI inhibitor D4476 for 10 h before collection. (**f**) IB analysis of HeLa cells treated with the CKI inhibitor D4476 and IC261 at the indicated concentrations for 12 h. (**g**) HeLa cells were infected with the shRNA constructs for GFP, or CKIδ followed by selection with 1 μg ml^−1^ puromycin for 3 days to eliminate non-infected cells. The generated stable cell lines were then split into 60-mm dishes. Cells were treated with 20 μg ml^−1^ CHX after 20 h. At the indicated time points, WCL were prepared and immunoblots were probed with the indicated antibodies. (**h**) Quantification of the Set8 band intensities in **g**. Set8 band intensity was normalized to tubulin, and then normalized to the *t*=0 controls.

Emerging evidence has demonstrated that one kinase or a combination of multiple kinases are required to properly phosphorylate substrates within their phosphodegron before substrate ubiquitination and degradation by the SCF type of E3 ligases including SCF^β-TRCP^ (refs 13, 23). To this end, it has been previously reported that CKI is often involved in β-TRCP-mediated protein degradation. Therefore, we sought to explore whether CKI is the upstream kinase that phosphorylates Set8 and subsequently triggering its destruction by SCF^β-TRCP^. Notably, we observed that both CKIδ and CKIα specifically interacted with Set8 (Fig. 3a). Moreover, depletion of endogenous *CKI*δ, and to a lesser extent, depletion of *CKIα*, but not *CKIɛ*, led to an accumulation of Set8 (Fig. 3b). Conversely, ectopic expression of either CKIδ or CKIα promoted the destruction of ectopically expressed Set8 (Fig. 3c). Consistently, degradation of Set8 was blocked after MG132 treatment (Fig. 3c), suggesting that the 26S proteasome is involved in this process. In further support of a physiological role for CKI in Set8 stability control, inactivating CKI by either depletion of *CKI*δ by shRNA or using a CKI inhibitor, D4476, significantly disrupted the interaction between Set8 and β-TRCP1 (Fig. 3d,e). Consistently, pharmacological inactivation of CKI by D4476 or IC261 led to an elevation of Set8 abundance in a dose-dependent manner (Fig. 3f and Supplementary Fig. 3a–d). More importantly, we demonstrated that depletion of endogenous *CKI*δ extended the Set8 protein half-life (Fig. 3g,h), whereas conversely, ectopic expression of CKIδ led to a reduction in the half-life of endogenous Set8 (Supplementary Fig. 3e,f). These results coherently support the notion that CKI is critically involved in the destruction of Set8 by SCF^β-TRCP^.

### CKIδ-mediated phosphorylation of Set8 at the Ser253 site

**Figure 4:**
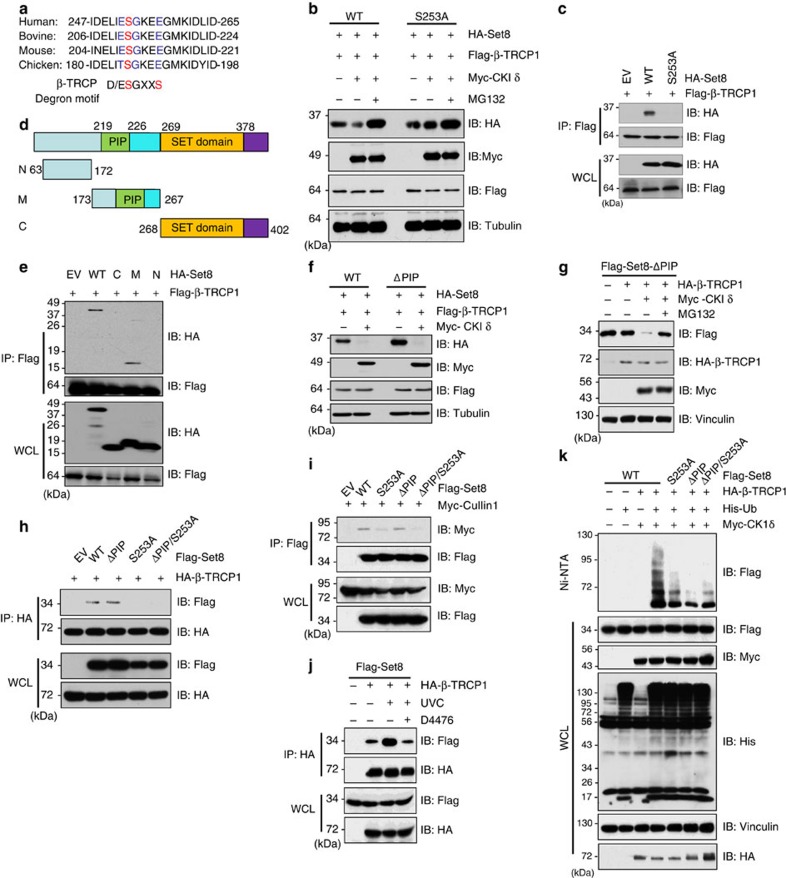
CKIδ phosphorylates Set8 at S253 site. (**a**) Alignment of the candidate phosphodegron sequence in Set8 from different species. (**b**) Immunoblot (IB) analysis of cells transfected with Flag-β-TRCP1 and HA-tagged wild type or the S253A mutant Set8 constructs, as indicated. Where indicated, cells were treated with Myc-CKIδ, or the proteasome inhibitor MG132. (**c**) IB analysis of WCL and IP derived from HeLa cells transfected with Flag-β-TRCP1, HA-WT-Set8 and HA-S253A-Set8. (**d**) A schematic illustration of Set8 functional domains and the truncation mutants with different domains (C, M, N domain) that are used in **e**. (**e**) IB analysis of WCL and IP derived from HeLa cells transfected with Flag-β-TRCP1, HA-WT-Set8 and HA-Set8 constructs with different domains (C, M, N domain). (**f**) IB analysis of HeLa cells transfected with Flag-β-TRCP1, Myc-CKIδ and Flag-tagged wild-type or ΔPIP mutant Set8 constructs, as indicated. (**g**) IB analysis of HeLa cells transfected with HA-β-TRCP1, Myc-CKIδ and Flag-tagged ΔPIP mutant Set8 constructs. Where indicated, 10 μM MG132 was added for 12 h before collecting. (**h**) IB analysis of WCL and IP derived from HeLa cells transfected with HA-β-TRCP1 and indicated Flag-Set8 constructs. (**i**) IB analysis of WCL and IP derived from 293 T cells transfected with Flag-Set8 mutants and Myc-tagged Cullin1. (**j**) IB analysis of WCL and IP derived from 293 T cells transfected with Flag-Set8 and HA-β-TRCP1 and treated with D4476 under UVC exposure condition. (**k**) *In vivo* ubiquitination assays to demonstrate that SCF^β-TRCP^ promotes Set8 ubiquitination in a pSer253-dependent manner. His-pull-downed ubiquitinated Set8 and WCL were subjected to IB analysis with indicated antibodies.

It has been reported by multiple groups that most β-TRCP substrates contain the canonical D/ESGxxS degron sequence^13^,^21^,^24^. Indeed, we observed that Set8 contains a putative ESGxxE degron that is conserved among various species (Fig. 4a). To further determine whether this putative phosphodegron is important for Set8 degradation, we mutated the Ser253 residue to alanine (thereafter termed the S253A mutant). Notably, *in vivo* degradation assays revealed that ectopic expression of β-TRCP1 and CKIδ led to a rapid disappearance of wild-type (WT), but not the S253A mutant form of, Set8 that is deficient in associating with β-TRCP1 (Fig. 4b,c and Supplementary Fig. 4a,b). Notably, MG132 prevented the degradation of Set8 (Fig. 4b and Supplementary Fig. 4a), indicating the involvement of the 26S proteasome pathway in mediating Set8 degradation by SCF^β-TRCP^. Moreover, co-immunoprecipitation experiments demonstrated that β-TRCP1 bound the middle domain of Set8 that contains the identified phosphodegron motif (Fig. 4d,e), further supporting a critical role for Ser253 in mediating β-TRCP1 interaction with Set8 in a phosphorylation-dependent manner (Fig. 1c).

Interestingly, the Set8^ΔPIP^ mutant that lacks the CRL4^Cdt2^ recognizable degron motif, thereby deficient in associating with Cullin 4A (ref. 9), could still be efficiently degraded by co-expression of CKIδ and β-TRCP (Fig. 4f), a process that can be blocked by MG132 treatment (Fig. 4g). Furthermore, unlike Set8^WT^ and Set8^ΔPIP^, Set8^S253A^ and Set8^ΔPIP/S253A^ were deficient in binding β-TRCP1 (Fig. 4h and Supplementary Fig. 4c), supporting the notion that SCF^β-TRCP^ regulates Set8 stability independent of CRL4^Cdt2^ and the PIP motif. Moreover, point mutation of the β-TRCP recognition motif of Set8, namely the Ser253 residue, but not the PIP motif, abolished the association between Set8 and Cullin 1 (Fig. 4i), as well as the interaction between Set8 and other SCF components such as Rbx1 (Supplementary Fig. 4d) and Skp1 (Supplementary Fig. 4e). More importantly, we showed that β-TRCP1 promoted the *in vitro* ubiquitination of WT-, but not the S253A mutant form of, Set8, in a CKIδ-dependent manner (Supplementary Fig. 4f). This result further suggests that the inefficient destruction of S253A by SCF^β-TRCP^ (Fig. 4b) could be likely due to deficient ubiquitination of Set8. Notably, ultraviolet irradiation augmented the interaction between Set8 and β-TRCP1, which was impeded by the treatment of CKI inhibitor D4476 (Fig. 4j), supporting a critical role for both SCF^β-TRCP^ and CKI in ultraviolet-induced degradation of Set8. Consistently, *in vivo* ubiquitination assays further demonstrated that SCF^β-TRCP^ promoted Set8 poly-ubiquitination in a Ser253-dependent manner (Fig. 4k and Supplementary Fig. 4g). Importantly, using mass spectrometry analysis, we identified Ser253 phosphorylation in cells (Supplementary Fig. 4h). Furthermore, the Ser253 residue is predicted to be a putative CKI site, which prefers D/ExxS or D/ExxxS consensus sequence 21,24. Taken together, these findings suggest that CKIδ could phosphorylate Set8 at S253 to trigger Set8 destruction.

### Effects of β-TRCP-mediated Set8 degradation on cell growth

**Figure 5:**
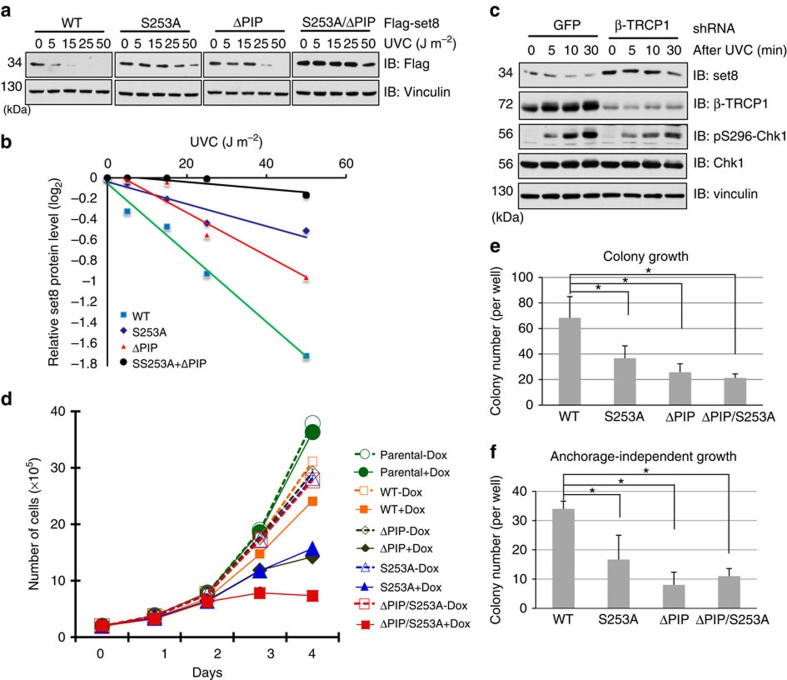
Effects of β-TRCP-mediated degradation of Set8 on cell growth. (**a**) U2OS cells with induced Flag-Set8^WT^, Set8^ΔPIP^, Set8^S253A^ or Set8^ΔPIP/S253A^ were treated with different doses of UVC (0–50 J m^−2^) for 10 min before whole cell lysates (WCL) were collected for immunoblot (IB) analysis to monitor the changes in Flag-Set8 abundance. (**b**) Quantification of the Flag-Set8 band intensities in **a**. Set8 band intensity was normalized to vinculin. (**c**) IB analysis of WCL from U2OS cells infected with lentivired shRNAs specific for GFP or β-TRCP1, treated with UVC (10 J m^−2^) and collected at indicated time points. (**d**–**f**) U2OS cells harbouring Flag-Set8^WT^, Set8^ΔPIP^, Set8^S253A^ or Set8^ΔPIP/S253A^ were cultured in the absence or presence of 0.1 μg ml^−1^ doxycycline (Dox). The total cell numbers at the different days were plotted (**d**). Clonogenic assay (**e**) and soft agar assay (**f**) were used to measure cell proliferation. Data are shown as mean±s.d. of three independent experiments. *P<0.05, Student's *t*-test.

It has been previously reported that Set8 protein levels were significantly reduced after DNA damage induced by ultraviolet C (UVC) treatment^8^,^9^. Consistent with these findings, we found that UVC could promote the degradation of ectopically expressed WT-Set8 in a dose-dependent manner (Fig. 5a). In support of a critical role of β-TRCP in UVC-induced degradation of Set8, we found that mutating the critical Ser253 residue within the β-TRCP recognizable degron motif, or depleting endogenous β-TRCP1, largely abolished ultraviolet-induced reduction of Set8 abundance, in part due to extended Set8 protein half-life (Fig. 5a–c and Supplementary Fig. 5a–c). However, we still observed a reduction of S253A-Set8 abundance on high-dose UVC treatment (25 and 50 J m^−2^; Fig. 5a,b).

Importantly, previous studies have also shown that ultraviolet-induced Set8 degradation is in part dependent on its PIP degron^9^. In line with this concept, we found that the Set8^ΔPIP^ mutant is more stable than Set8^WT^ after UVC treatment (Fig. 5a,b and Supplementary Fig. 5b,c). However, the abundance of the Set8^ΔPIP^ mutant is also reduced in relatively high-dose UVC treatment (25 and 50 J m^−2^; Fig. 5a,b). On the other hand, simultaneous inactivation of both degrons created a mutant version of Set8 that are more resistant to UVC-induced degradation, even in high dosage of UVC treatment (Fig. 5a,b). These data suggest the possibility that both Cdt2 and β-TRCP have non-redundant roles in governing UVC-induced Set8 ubiquitination and degradation, such that simultaneous inactivation of both CRL4^Cdt2^- and SCF^β-TRCP^-mediated pathways are required to abolish UVC-induced degradation of Set8 in cells.

Furthermore, given its critical role in epigenetic and cell cycle regulation, previous studies have revealed that forced Set8 expression slowed down cell proliferation^9^. Consistent with this report, we found that ectopic expression of Set8^WT^ inhibited cell proliferation. Moreover, ectopic expression of the relatively more stable version of the Set8^ΔPIP^ and Set8^S253A^ mutants caused a more marked effect in inhibiting cell proliferation, compared with expression of Set8^WT^ (Fig. 5d). More importantly, ectopic expression of the Set8^ΔPIP/S253A^ mutant, which is much more resistant to UVC-induced degradation (Supplementary Fig. 5b,c), led to a greater inhibition of cell proliferation compared with the expression of Set8^WT^, Set8^ΔPIP^ or Set8^S253A^ alone (Fig. 5d and Supplementary Fig. 5d). Moreover, experimental results derived from colony formation assays further validated that Set8^ΔPIP^ and Set8^S253A^ are more potent than Set8^WT^ in suppressing colony formation *in vitro* (Fig. 5e). Interestingly, disrupting both CRL4^Cdt2^- and SCF^β-TRCP^-mediated pathways allows the Set8^ΔPIP/S253A^ double mutant to be the most potent among all Set8 constructs we examined to inhibit colony growth, but not the anchorage-independent growth, *in vitro* (Fig. 5e,f). These results revealed both CRL4^Cdt2^ and SCF^β-TRCP^ as part of the degradation network to govern the timely destruction of Set8, and further suggest that aberrancies in these destruction pathways will likely cause adverse effects, leading to aberrant cell cycle progression or chromosome instability.

### β-TRCP-mediated Set8 degradation affects cell cycle

**Figure 6:**
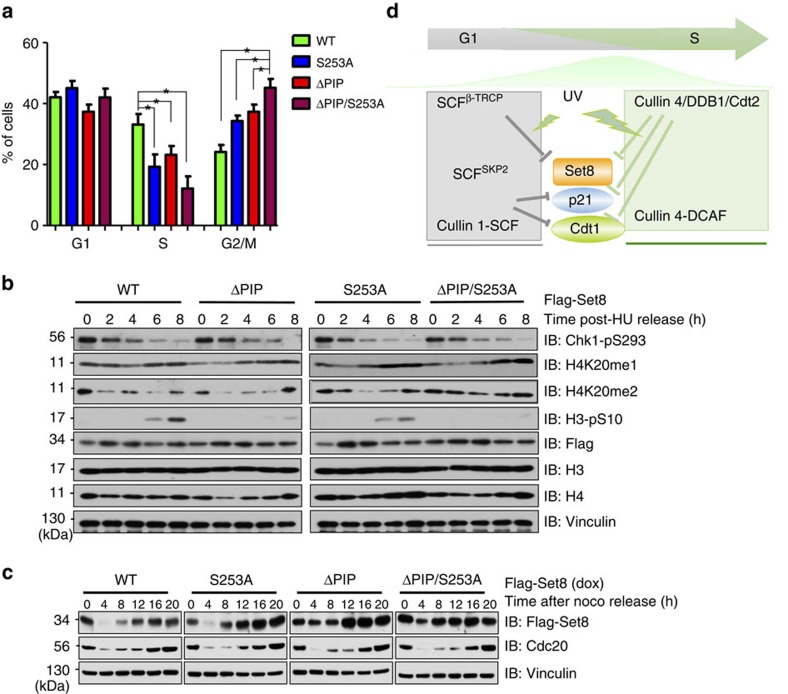
Effects of β-TRCP-mediated degradation of Set8 on cell cycle progression. (**a**) U2OS cells harbouring Flag-Set8^WT^, Set8^ΔPIP^, Set8^S253A^ or Set8^ΔPIP/S253A^ were cultured in the presence of 0.1 μg ml^−1^ doxycycline (Dox). Cell cycle was analysed by propidium iodide staining using fluorescence-activated cell sorting. Data are shown as mean±s.d. of three independent experiments. *P<0.05, Student's *t*-test. (**b**) U2OS cells harbouring inducible Flag-tagged Set8^WT^, Set8^ΔPIP^, Set8^S253A^ or Set8^ΔPIP/S253A^ were synchronized in S phase with hydroxyurea (HU) for 24 h. During the last 4 h in HU, expression of Set8 was induced by Dox. Then, cells were released from HU in the presence of Dox, and IB analysis was performed to detect the indicated proteins. (**c**) U2OS cells harbouring inducible Flag-tagged Set8^WT^, Set8^ΔPIP^, Set8^S253A^ or Set8^ΔPIP/S253A^ were synchronized in M phase with nocodazole for 18 h. During the last 4 h in nocodazole, expression of Set8 was induced by Dox. Then, cells were released from nocodazole in the presence of Dox, and IB analysis was performed to detect the indicated proteins. (**d**) A schematic illustration of the signalling network that timely regulates the Set8 protein stability during the cell cycle progression.

It has been reported that both Set8 expression levels and H4K20me1 intensities fluctuate during the cell cycle, and Set8-mediated H4K20me plays a critical role in regulating cell cycle^8^,^9^,^25^. Consistent with a previous report^9^, we found that Dox-induced expression of Set8^ΔPIP^ caused a prominent increase in G2/M cell populations compared with Set8^WT^ (Fig. 6a and Supplementary Fig. 6a–h). Moreover, Set8^S253A^ also caused a robust G2/M cell cycle arrest compared with Set8^WT^. Interestingly, the Set8^ΔPIP/S253A^ mutant only led to a moderately increased G2/M arrest compared with Set8^ΔPIP^ (Fig. 6a). These results indicate that further investigation is required to explore the role of each of these degradation pathways in regulating Set8-mediated G2/M arrest.

Mechanistically, previous studies have revealed that ectopic expression of the Set8^ΔPIP^ mutant induced H4K20me1 accumulation and phospho-Chk1 to cause the cell cycle defects^9^. To further determine the mechanism of β-TRCP-mediated degradation of Set8 in regulation of cell cycle, we monitored the changes of pChk1, H4K20me1, H4K20me2, H4K20me3 and pSer10-H3 in cells ectopically expressing Set8^WT^, Set8^ΔPIP^, Set8^S253A^ or Set8^ΔPIP/S253A^. Interestingly, we found that cells released from a late G1/S phase arrest after hydroxyurea (HU) challenge, ectopic expression of WT-Set8 only minimally affected cell cycle progression as it can be degraded in G2/M phase and cells enter M phase in around 6–8 h post-HU release, as evidenced by elevation of pSer10-H3. In keeping with previous reports^9^, expressing the more stabilized versions of Set8^ΔPIP^ or Set8^S253A^, led to a retardation of cell cycle progression, delaying cells to enter M phase, as evidenced by reduced pSer10-H3 in 6–8 h time points. Furthermore, consistent with the robust potencies in arresting cells in G2/M phase, Dox-induced expression of the Set8^ΔPIP/S253A^ mutant led to a more dramatic delay for cells to enter M phase, as there were only residual pSer10-H3 signals detected 8 h post-HU release (Fig. 6b). Consistent with an elevated methyltransferase activity, there were more H4K20me2 signals detected in the 8 h time point of Set8^ΔPIP/S253A^-expressing cells (Fig. 6b and Supplementary Fig. 6i,j). However, further studies are required to make the molecular connection between elevation of H4K20me2 and increased activity of the p53/p21 tumor-suppressor pathway to functionally induce G2/M cell cycle arrest. Furthermore, in support of a critical role of both CRL4^Cdt2^ and/or SCF^β-TRCP^-mediated Set8 degradation pathways in governing Set8 activity/abundance during the cell cycle progression, we observed that compared with WT-Set8, Set8^S253A^ was more stable in the G1 phase (8 h post release), while the Set8^ΔPIP^ was relatively more stable as cells enter into S phase (12–16 h post release) (Fig. 6c and Supplementary Fig. 6k). Notably, the Set8^ΔPIP/S253A^ double mutant may evade both degradation pathways, thereby becoming stabilized in both G1 and S phases (8–16 time points) (Fig. 6c). These results further support the notion that like many other cell cycle regulators including p21 and Cdt1 (refs 26, 27), Set8 stability is likely controlled by different sets of E3 ligases including CRL4^Cdt2^ and/or SCF^β-TRCP^ that may function in different cell cycle phases to timely control the abundance of Set8 (Fig. 6d).

## Discussion

Previous studies have reported that CRL4^Cdt2^ (refs 4, 8, 9) and APC^Cdh1^ (ref. 7) promote the degradation of Set8 during cell cycle, indicating that Set8 is required to be maintained at precise levels by multiple E3 ligases for proper cell cycle progression. Here, we report SCF^β-TRCP^ as another E3 ligase that controls Set8 destruction to govern cell cycle transition. Different from other studies, we discovered CKIδ as the upstream kinase that phosphorylates Set8 at Ser253 to trigger its ubiquitination and subsequent degradation by SCF^β-TRCP^. In line with this finding, Set8^WT^, but not Set8^S253A^, interacted with β-TRCP. Moreover, Set8^S253A^ displayed extended half-life in β-TRCP/CKIδ-mediated degradation.Taken together, our data suggests a critical role for Ser253 phosphorylation in controlling Set8 stability. Since Set8 is an important cell cycle regulator, regulation of β-TRCP and CKIδ could govern proper cell cycle progression through control of Set8 stability. More importantly, as CKIδ has been reported by multiple groups to be activated by the DNA damage signals^24^,^28^,^29^, our results provide an important molecular link between DNA damage-induced kinase cascade and the timely destruction of Set8 (Supplementary Fig. 7).

It has been previously identified that CRL4^Cdt2^ is responsible for Set8 proteolytic degradation in the S phase of the cell cycle^8^,^12^,^30^. Specifically, CRL4^Cdt2^ promotes the degradation of Set8, which is dependent on the Set8–PCNA interaction, leading to attenuated levels of H4K20me1 during S phase^8^. In S phase and after DNA damage, CRL4^Cdt2^ promotes the destruction of both Set8 and Cdt1 (ref. 9). The PIP degron of Set8 is required for this process. In support of this notion, we observed that Set8^ΔPIP^ induced greater G2/M arrest compared with Set8^WT^ (Fig. 6a and Supplementary Fig. 6a–h). Similarly, Set8^S253A^ also caused a robust G2 arrest, indicating that β-TRCP-mediated Set8 degradation is also involved in regulating cell cycle progression. Multiple lines of evidence have indicated that inactivation of the CRL4^Cdt2^-mediated Set8 degradation inhibits cell proliferation^8^,^9^. In line with this, Set8^S253A^, phenocopying Set8^ΔPIP^, led to a marked inhibition in cell proliferation (Fig. 5d), suggesting that like CRL4^Cdt2^, β-TRCP-mediated Set8 destruction also plays an essential role in cell proliferation. Furthermore, we obtained evidence from both biochemical and cellular assays showing that CRL4^Cdt2^ and SCF^β-TRCP^ are likely independent pathways in governing Set8 stability. As such, simultaneous inactivation of both destruction mechanisms led to a more resistant mutant form of Set8 (Set8^ΔPIP/S253A^) that is more potent in inhibiting cell growth.

Recent studies have also highlighted the important role of Set8 in tumorigenesis. Specifically, Set8 expression is positively associated with metastasis in breast cancer patients^31^. Several studies have demonstrated that patients with low Set8 levels have longer survival in hepatocellular carcinoma^32^ and small-cell lung cancer^33^. In addition, low expression of Set8 contributes to a decreased risk of epithelial ovarian cancer^34^. Recently, Set8 was found to be involved in the androgen receptor (AR)-mediated transcription activation via interaction with AR and H4K20me1 in prostate cancer^35^. Overexpression of Set8 promoted AR-induced cell proliferation in prostate cancer cells^35^, suggesting that the function of Set8 in regulating cell proliferation remains largely controversial. More importantly, besides cell proliferation, Set8 was found to promote epithelial–mesenchymal transition and invasion in breast cancer cells through interplay with Twist via its dual chromatin remodelling activity^31^. Consistently, downregulation of Set8 by the tumour suppressor miRNA-7 inhibited H4K20me1, leading to suppression of epithelial–mesenchymal transition and metastasis of breast cancer cells, and sensitizing cells to DNA damages^36^. Altogether, these clinical studies suggest that Set8 largely functions as an oncogene, but might be primarily associated with late events such as cancer metastasis.

It is important to note that recent studies from multiple groups showed that acute expression of an oncogene such as Ha-Ras^37^, Akt^38^,^39^, BRAF^40^,^41^ or hypoxia-inducible factor 1 (HIF1)^42^, would rather lead to a growth arrest phenotype, but not accelerated cell proliferation. This feature is specifically termed OIS, in short for oncogene-induced senescence. In support of this notion, genetic deletion of phosphatase and tensin homolog (PTEN) deleted on chromosome 10 (refs 43, 44) or von hippel-lindau (VHL)^45^ tumour suppressor also led to the onset of senescence, likely due to aberrant expression of pAkt and HIF oncoproteins, while ectopic expression of BRAF directly led to senescence in melanocytes^40^. We speculate that similarly, stabilization of the Set8 oncoprotein by disrupting either the CRL4^Cdt2^ or the SCF^β-TRCP^ pathway might cause growth arrest (Figs 5d and 6a–c) through the OIS mechanism. Given that β-TRCP-mediated degradation of Set8 is in a CKIδ-dependent manner, modulation of β-TRCP and/or CKIδ may be beneficial in treating a variety of human cancers.

Collectively, our results provide mechanistic insights into a novel degradation pathway for Set8 during cell cycle transition. It is possible that like many other critical cell cycle regulators including p21 and Cdt2, the stability of Set8 is tightly and synergistically regulated by multiple different E3 ligases (Fig. 6d). In S phase, Set8 destruction is primarily promoted by CRL4^Cdt2^, while in G1 phase, Set8 can be controlled by Skp2 and β-TRCP in different cellular context, with the β-TRCP/CKI pathway devotes to DNA damage-induced Set8 destruction (Fig. 6d). Without a doubt, further investigations are required to decipher how the orchestration of three E3 ligases controls the timely degradation of Set8 to govern proper cell cycle progression. Given that Set8 is critically involved in cell cycle progression, our studies could provide a possible mechanism for dysregulated cell cycle in cancer and further suggest that targeting β-TRCP could be a promising approach for the treatment of human cancers.

## Methods

### Cell culture

293T, HeLa, T98G and U2OS cells were obtained from the American Type Culture Collection (ATCC) and cultured in DMEM medium (Life Technologies, CA, USA) supplemented with 10% fetal bovine serum, penicillin and streptomycin. The cells were maintained in a 5% CO_2_-humidified atmosphere at 37 °C. *Cullin 4A*^*fl/fl*^ (ref. 46) and *Cullin 4B*^*fl/fl*^ (ref. 47) mouse embryonic fibroblasts were kindly provided and characterized by Dr Pengbo Zhou (Weill Cornell Medical College, New York, NY, USA).

### Plasmids

Various Set8 mutants were generated using the QuikChange XL Site-Directed Mutagenesis Kit (Stratagene) according to the manufacturer's instructions. Human siRNA oligos against Skp2 (A-sense, 5′-AAGGUCUCUGGUGUUUGUAAG-3′; B-sense, 5′-AAGCAUGUACAGGUGGCUGUU-3′), β-TRCP1+2 (sense, 5′-AAGUGGAAUUUGUGGAACAUC-3′) and Cdh1 (sense, 5′-UGAGAAGUCUCCCAGUCAGUU-3′) were purchased from Dharmacon. Short hairpin RNAs (shRNA lentivirus vectors), including shRNA-β-TRCP1, shRNA-β-TRCP1+2, shRNA-GFP, Flag-β-TRCP1, Flag-β-TRCP1-R474A constructs, and CKI constructs were obtained from Dr Wade Harper (Harvard Medical School, Boston, MA, USA). shRNA lentiviral vectors against Cullin 1 were gifts from J. Wade Harper (Harvard Medical School). Myc-Cullin 1, Myc-Cullin 2, Myc-Cullin 3, Myc-Cullin 4 and Myc-Cullin 5 constructs were obtained from James DeCaprio (Dana-Farber Cancer Institute, Boston, MA, USA). Lentiviral shRNA constructs against green fluorescent protein (GFP) and various CKI isoforms were gifts from William Hahn (Dana-Farber Cancer Institute, Boston, MA, USA). Lentiviral shRNA constructs against Cullin 4A and Cullin 4B were purchased from Open Biosystems (Lafayette, CO, USA).

### Antibodies and reagents

Anti-Set8 (C18B7; 1:1,000 immunoblot (IB)), anti-β-catenin (D10A8; 1:1,000 IB), anti-Skp2 (D3G5; 1:1,000 IB), anti-Mcl-1 (D35A5; 1:1,000 IB), anti-G9A (C6H3; 1:1,000 IB), anti-Cullin 1 (4995; 1:1,000 IB) and anti-β-TRCP1 (4394; 1:1,000 IB) antibodies were purchased from Cell Signaling Technology. Anti-p27 (SC-528; 1:500 IB), anti-Cullin 1 (SC-17775; 1:1,000 IB), anti-casein kinase 1δ (H-60; 1:1,000 IB), anti-casein kinase 1α (SC-6474; 1:1,000 IB), anti-c-Myc (9E10; 1:1,000 IB, 2 μg immunoprecipitates (IP)) and polyclonal anti-HA antibodies (SC-805; 1:1,000 IB, 2 μg IP) were purchased from Santa Cruz Biotechnology. Anti-Vinculin antibody (V-4505; 1:2,000 IB), monoclonal anti-Flag antibody (F-3165), polyclonal anti-Flag antibody (F-2425; 1:1,000 IB, 2 μg IP), anti-HA agarose beads (A-2095; 1:1,000 IB, 2 μg IP), peroxidase-conjugated anti-mouse secondary antibody (A-4416; 1:3,000 IB) and peroxidase-conjugated anti-rabbit secondary antibody (A-4914; 1:3,000 IB) were purchased from Sigma. Monoclonal anti-HA antibody (MMS-101P) was purchased from Covance. Anti-GFP antibody (632380; 1:1,000 IB), anti-Cdh1 (34-2000; 1:1,000 IB) antibody, Oligofectamine, Lipofectamine and Plus reagents were purchased from Invitrogen. Anti-CUL 4B (16184-1-AP; 1:1,000 IB) antibody was purchased from Proteintech Group.

### Immunoblots and immunoprecipitation

Cells were lysed in EBC lysis buffer (50 mM Tris pH 8.0, 120 mM NaCl, 0.5% NP-40) supplemented with protease inhibitors (Roche) and phosphatase inhibitors (EMD Millipore). The protein concentrations were measured using the Bio-Rad protein assay reagent (Bio-Rad Laboratories, CA, USA). The lysates were then resolved by SDS–PAGE and immunoblotted with indicated antibodies as described before^48^. For immunoprecipitation assays, 800 μg of protein lysates were incubated with the appropriate antibody (1–2 μg) overnight at 4 °C followed by addition of carrier beads for 1 h. Immunocomplexes were washed with NETN buffer (20 mM Tris, pH 8.0, 100 mM NaCl, 1 mM EDTA and 0.5% NP-40), then resolved by SDS–PAGE and immunoblotted with indicated antibodies. Quantification of the immunoblot band intensity was performed with ImageJ software. Full scans of IBs are presented in Supplementary Fig. 8.

### Mass spectrometry analysis

Whole cellular extracts from Flag-β-TRCP1 stably expressing 293 T cells were subjected to affinity purification with anti-Flag antibody. The purified protein complex was resolved by SDS–PAGE and silver stained, and the bands were retrieved and analysed by mass spectrometry.

### Real-time RT–PCR analysis

RNA was extracted using Qiagen RNeasy mini kit, and the reverse transcription reaction was performed using the ABI Taqman Reverse Transcriptional Reagents. After mixing the resulting template with β-TRCP2 (Hs00362667_m1) or GAPDH (Hs99999905_m1) primers and ABI Taqman Fast Universal PCR Master Mix, the real-time reverse transcription (RT)–PCR reaction was performed with the ABI-7500 Fast Real-time PCR system

### Protein degradation analysis

Cells were transfected with a plasmid encoding a HA-tagged version of Set8 along with Flag-β-TRCP1, and a plasmid encoding GFP as an internal control, in the presence or absence of Myc-CKIδ. After 40 h, cells were lysed and subsequently immunoblot analysis was performed. For half-life studies, 20 μg ml^−1^ cycloheximide (CHX, Sigma) was added to the medium 40 h post transfection. At various time points thereafter, cells were lysed and immunoblot analysis was conducted to detect protein abundances.

### *In vitro* ubiquitination assay

The *in vitro* ubiquitination assays were conducted. The SCF^β-TRCP1^ (E3) complexes were purified from cells transfected with vectors encoding GST-β-TRCP1, Myc-Cul-1, Myc-Skp1 and HA-Rbx1. The indicated GST-Set8 (amino acids 173–267) proteins were incubated with purified, recombinant active CKIδ in the presence of ATP at 30 °C for 30 min. Then, the kinase reaction products were incubated with SCF^β-TRCP1^ (E3) complexes in the presence of purified, recombinant active E1, E2, ATP, and ubiquitin at 30 °C for 45 min. The immunoblot analysis was performed using the indicated antibodies.

### Cell transfection

For cell transfection, cells were seeded in 60 mm plates and transfected using Lipofectamine 2000 (Invitrogen) in OptiMEM medium (Invitrogen) for 48 h according to the manufacturer's instructions. Real-time RT–PCR or western blot analysis was used to detect the efficacy of transfection.

### Cell cycle and colony formation and soft agar assay

Cells were collected, fixed with 75% ethanol and stained with propidium iodide for flow cytometry analysis to assess cell cycle. Cells were seeded into the gelatinized plates. After 4 days, the colonies were stained on the plates with crystal violet and counted. The numbers of surviving colonies were calculated as the average of triplicates. Two percent melting point agar was prepared and mixed with RPMI1640 to make the 0.4 and 0.8% agar in 50 °C. Two millilitre 0.8% agar was added in the bottom of the six-well plate. About 3 × 10^4^ cells and 2 ml 0.4% agar were mixed, and the mixture was added on the top of 0.8% agar. After routine culture for 2 weeks, colony numbers were counted under a microscope.

### Statistical analysis

The significance of the data between experimental groups was determined by Student's *t*-test. *P*<0.05 was considered to be significant.

## Additional information

**How to cite this article:** Wang, Z. *et al.* SCF^β-TRCP^ promotes cell growth by targeting PR-Set7/Set8 for degradation. *Nat. Commun.* 6:10185 doi: 10.1038/ncomms10185 (2015).

## Supplementary Material

Supplementary Information Supplementary Figures 1-8
