# Supplementary material for: SCFβ-TRCP promotes cell growth by targeting PR-Set7/Set8 for degradation
Source: Nat Commun. 2015 Dec 15;6:10185. doi: 10.1038/ncomms10185 (PMC4682171; doi:10.1038/ncomms10185)
Supplement: Supplementary Information — Supplementary Figures 1-8 [file ncomms10185-s1.pdf]

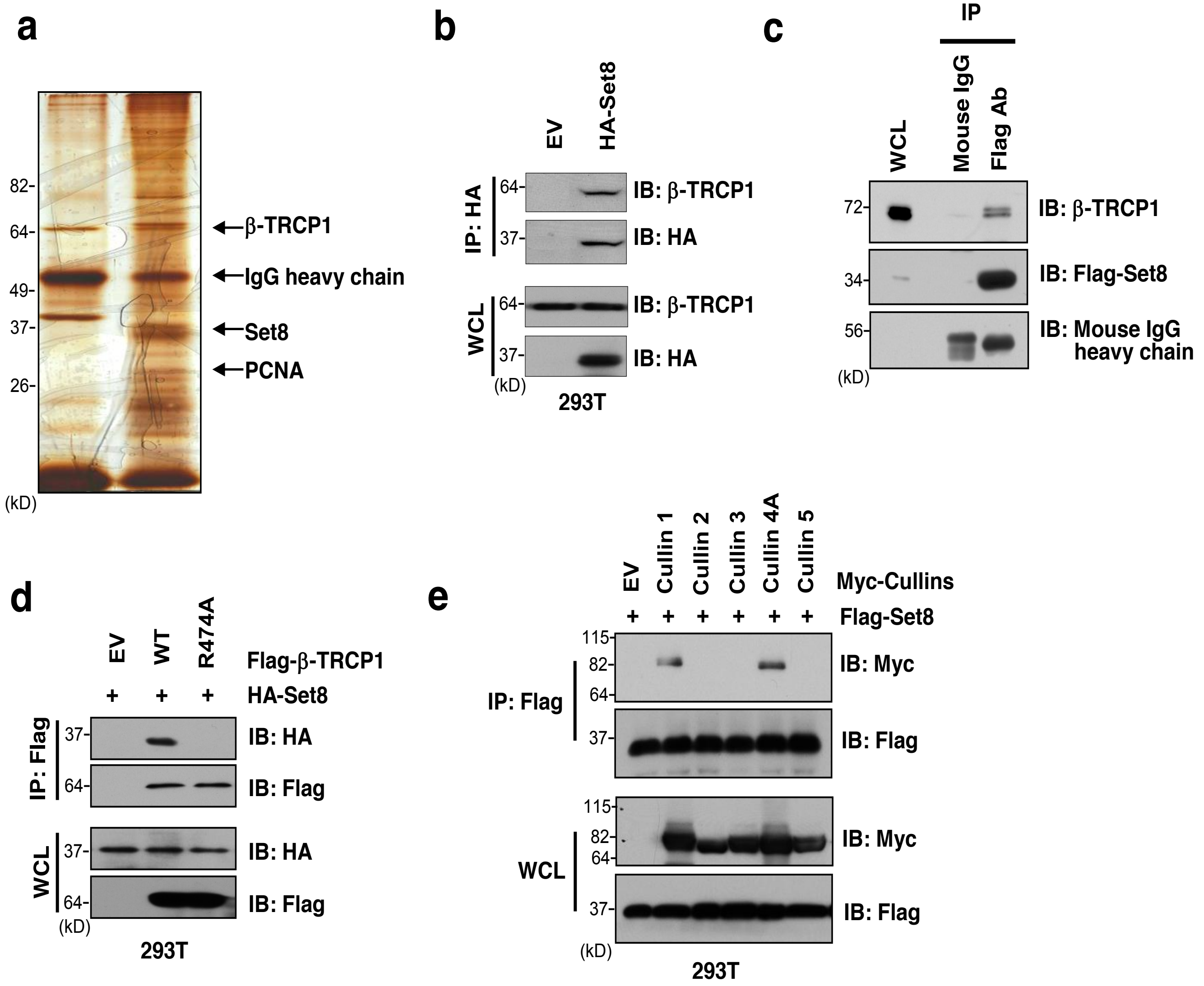

Supplementary Figure 1. Set8 interacts with the SCF$^{\beta\text{-TRCP}}$ complex.

a. Mass spectrometry analysis of β-TRCP1-associated proteins.
b. Immunoblot (IB) analysis of whole cell lysates (WCL) and immunoprecipitation (IP) derived from 293T cells transfected with HA-Set8 or empty vector (EV) as a negative control.
c. U2OS cells harboring inducible Flag-tagged Set8^{WT} were treated with 0.1 μg ml^{-1} doxycline (Dox) to induce the ectopic expression of Flag-WT-Set8. The cells were then treated with 15 μM MG132 for 12 hr and harvested for anti-Flag immunoprecipitation (IP). Mouse normal IgG was used as a control.
d. IB analysis of WCL and IP derived from 293T cells transfected with HA-Set8 and Flag-tagged wild-type (WT) or R474A mutant β-TRCP1 constructs, or EV.
e. IB analysis of WCL and IP derived from 293T cells transfected with Flag-Set8 and Myc-Cullin 1, 2, 3, 4A or 5 expressing plasmids.

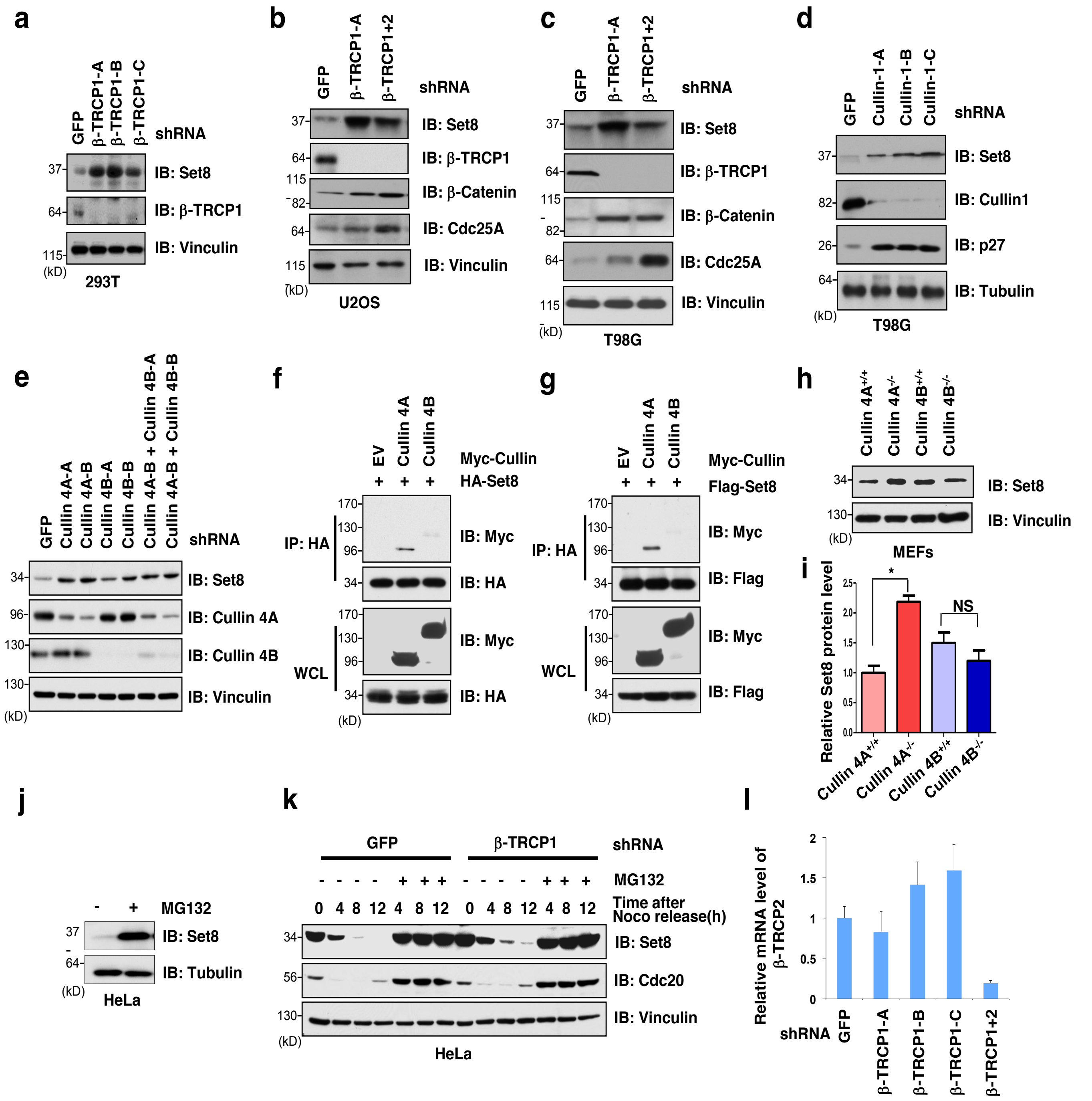

a
GFP
β-TRCP1-A
β-TRCP1-B
β-TRCP1-C
shRNA
IB: Set8
IB: β-TRCP1
IB: Vinculin
(kD)
293T
b
GFP
β-TRCP1-A
β-TRCP1+2
shRNA
IB: Set8
IB: β-TRCP1
IB: β-Catenin
IB: Cdc25A
IB: Vinculin
(kD)
U2OS
c
GFP
β-TRCP1-A
β-TRCP1+2
shRNA
IB: Set8
IB: β-TRCP1
IB: β-Catenin
IB: Cdc25A
IB: Vinculin
(kD)
T98G
d
GFP
Cullin-1-A
Cullin-1-B
Cullin-1-C
shRNA
IB: Set8
IB: Cullin1
IB: p27
IB: Tubulin
(kD)
T98G
e
GFP
Cullin 4A-A
Cullin 4A-B
Cullin 4B-A
Cullin 4B-B
Cullin 4A-B + Cullin 4B-A
Cullin 4A-B + Cullin 4B-B
shRNA
IB: Set8
IB: Cullin 4A
IB: Cullin 4B
IB: Vinculin
(kD)
f
EV
Cullin 4A
Cullin 4B
Myc-Cullin
HA-Set8
IP: HA
IB: Myc
IB: HA
WCL
IB: Myc
IB: HA
(kD)
g
EV
Cullin 4A
Cullin 4B
Myc-Cullin
Flag-Set8
IP: HA
IB: Myc
IB: Flag
WCL
IB: Myc
IB: Flag
(kD)
h
Cullin 4A+/+
Cullin 4A-/-
Cullin 4B+/+
Cullin 4B-/-
IB: Set8
IB: Vinculin
(kD)
MEFs
i
Relative Set8 protein level
*
NS
Cullin 4A+/+
Cullin 4A-/-
Cullin 4B+/+
Cullin 4B-/-
j
MG132
IB: Set8
IB: Tubulin
(kD)
HeLa
k
GFP
β-TRCP1
shRNA
MG132
Time after Noco release(h)
IB: Set8
IB: Cdc20
IB: Vinculin
(kD)
HeLa
l
Relative mRNA level of β-TRCP2
shRNA
GFP
β-TRCP1-A
β-TRCP1-B
β-TRCP1-C
β-TRCP1+2

Supplementary Figure 2. Depletion of endogenous *β-TRCP* or *Cullin 1* increases Set8 protein levels.

a-c. Immunoblot (IB) analysis of whole cell lysates (WCL) derived from 293T (**a**), U2OS (**b**), and T98G (**c**) infected with shRNA constructs specific for *GFP*, *β-TRCP1* (three independent shRNAs, namely –A, -B and -C), or *β-TRCP1+2*, followed by selection with 1 μg ml^{-1} puromycin for three days to eliminate non-infected cells.

d. IB analysis of WCL from T98G cells infected with shRNA specific for *GFP*, or several shRNA constructs against *Cullin 1* (three independent lentiviral Cullin 1-targeting shRNA constructs namely, -A, -B, -C) followed by selection with 1 μg ml^{-1} puromycin for three days to eliminate non-infected cells.

e. IB analysis of WCL from HeLa cells infected with shRNA construct specific for *GFP*, or several shRNA constructs against *Cullin 4* (two independent lentiviral *Cullin 4A*-targeting shRNA constructs namely, -A, -B, and two independent lentiviral *Cullin 4B*-targeting shRNA constructs namely, -A, -B) followed by selection with 1 μg ml^{-1} puromycin for three days to eliminate non-infected cells.

f. IB analysis of WCL and IP derived from 293T cells transfected with HA-Set8 and Myc-tagged Cullin 4A and Cullin 4B.

g. IB analysis of WCL and IP derived from 293T cells transfected with Flag-Set8 and Myc-tagged Cullin 4A and Cullin 4B.

h. IB analysis of WCL derived from *Cullin* $4A^{fl/fl}$ and *Cullin* $4B^{fl/fl}$ MEFs that are retrovirally infected with the Cre-recombinase encoding vectors to delete endogenous *Cullin 4A* or *Cullin 4B*, respectively.

i. Quantification of the Set8 band intensities in (**h**). Set8 band intensity was normalized to Vinculin. Data are shown as mean ± s.d. of three independent experiments. *$p<0.05$, Student's t-test

j. IB analysis of WCL derived from HeLa cells with or without MG132 treatment.

k. HeLa cells were infected with the shGFP or shβ-TRCP1 followed by selection with 1 μg ml^{-1} puromycin for three days to eliminate non-infected cells. The generated stable cell lines were then treated with nocodazole. At the indicated time points, WCL were prepared and immunoblots were probed with the indicated antibodies.

l. mRNA levels of *β-TRCP2* in HeLa cells infected with the indicated shRNA constructs. *β-TRCP2* mRNA levels were normalized to *GAPDH*, and then normalized to the control cells (shGFP), n=3.

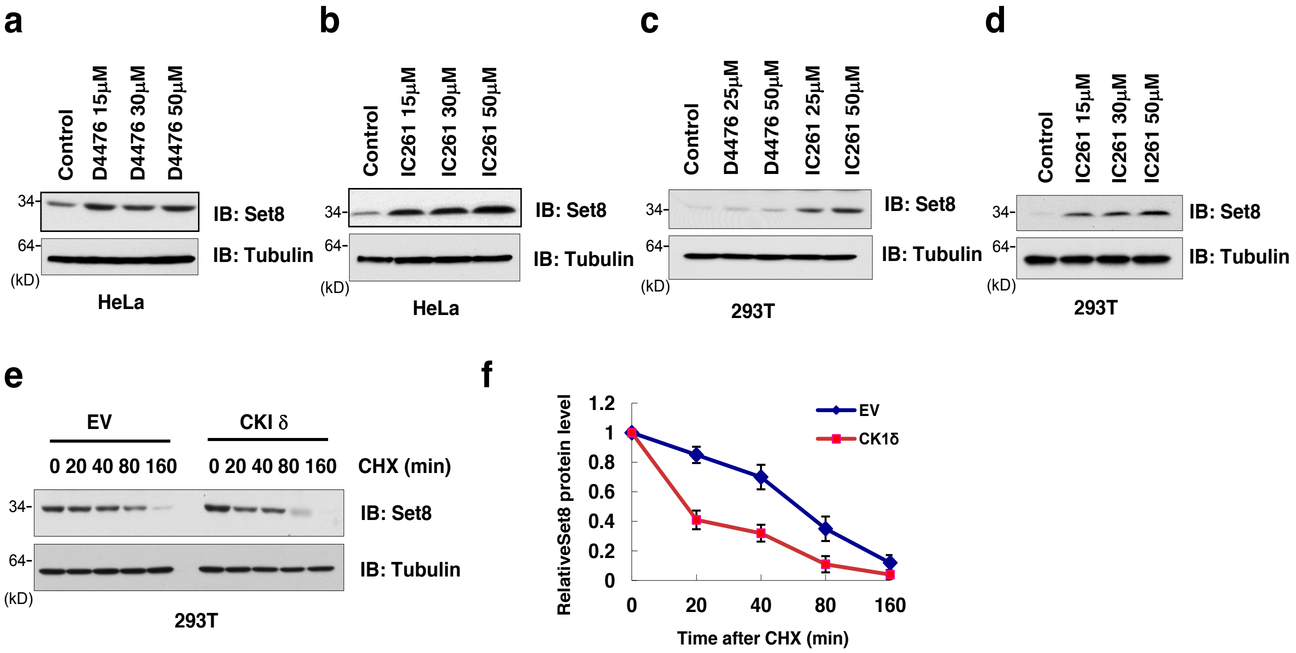

Supplementary Figure 3. Casein Kinase Iδ (CKIδ) controls Set8 stability.

a-b. Immunoblot (IB) analysis of WCL from HeLa cells treated with the CKI inhibitor D4476 (**a**) or IC261 (**b**) at the indicated concentrations for 12 hours.

c-d. IB analysis of WCL from 293T cells treated with the CKI inhibitor D4476 (**c**) or IC261 (**d**) at the indicated concentrations for 12 hours.

e. 293T cells were transfected with EV or CKIδ expression plasmid. The transfected cells were then split into 60-mm dishes. Cells were treated with 20 μg ml^{-1} CHX after 20 hours. At the indicated time points, WCL were prepared, and immunoblots were probed with the indicated antibodies.

f. Quantification of the Set8 band intensities in (**e**). Set8 band intensity was normalized to tubulin, and then normalized to the t=0 controls, n=3.

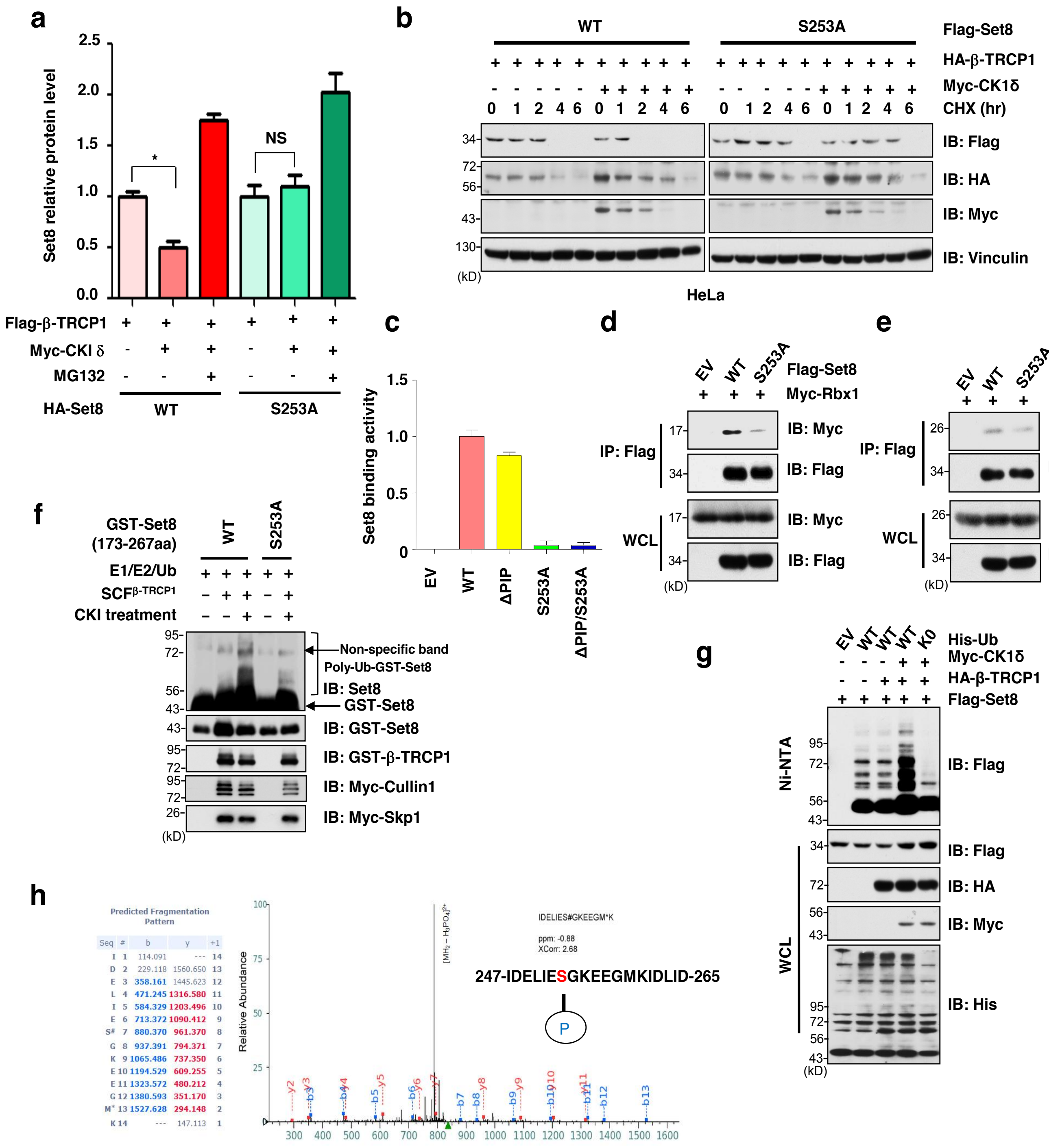
a
Set8 relative protein level
Flag-β-TRCP1
Myc-CKI δ
MG132
HA-Set8
WT
S253A
NS
b
WT
S253A
Flag-Set8
HA-β-TRCP1
Myc-CK1δ
CHX (hr)
IB: Flag
IB: HA
IB: Myc
IB: Vinculin
HeLa
c
Set8 binding activity
EV
WT
ΔPIP
S253A
ΔPIP/S253A
d
Flag-Set8
Myc-Rbx1
IP: Flag
WCL
IB: Myc
IB: Flag
e
Flag-Set8
Myc-Skp1
IP: Flag
WCL
IB: Myc
IB: Flag
f
GST-Set8 (173-267aa)
E1/E2/Ub
SCFβ-TRCP1
CKI treatment
Non-specific band
Poly-Ub-GST-Set8
IB: Set8
GST-Set8
IB: GST-Set8
IB: GST-β-TRCP1
IB: Myc-Cullin1
IB: Myc-Skp1
g
His-Ub
Myc-CK1δ
HA-β-TRCP1
Flag-Set8
Ni-NTA
WCL
IB: Flag
IB: HA
IB: Myc
IB: His
h
Predicted Fragmentation Pattern
Relative Abundance
IDELIES#GKEEGM*K
ppm: -0.88
XCorr: 2.68
247-IDELIESGKEEGMKIDLID-265
P

Supplementary Figure 4. Phosphorylation-dependent ubiquitination of Set8 by SCF$^{\beta\text{-TRCP}}$.

a. Quantification of the Set8 band intensities in Fig. 4b. Set8 band intensity was normalized to tubulin. Data are shown as mean ± s.d. of three independent experiments. $*p<0.05$, Student's t-test

b. HeLa cells were transfected with the indicated plasmids. The transfected cells were treated with 20 μg ml^{-1} CHX after 20 hours. At indicated time points, whole cell lysates (WCL) were prepared and the immunoblots were probed with the indicated antibodies.

c. Quantification of the Set8 band intensities in Fig. 4h. Set8 band intensity was normalized to Set8 in WCL,

d-e. IB analysis of WCL and immunoprecipitation (IP) derived from 293T cells transfected with Flag-Set8 and Myc-tagged Rbx1 or Skp1.

f. Purified CKIδ protein was incubated with 5 μg of the indicated GST-Set8 (173-267aa) proteins in the presence of ATP. The kinase reaction products were incubated with purified E1, E2, ubiquitin, and the SCF$^{\beta\text{-TRCP}}$ complex for 45 min. Afterwards, the products were analyzed by IB with anti-Set8 antibody.

g. *In vivo* ubiquitination assays to demonstrate that SCF$^{\beta\text{-TRCP}}$ promotes Set8 ubiquitination using the WT and K-0 mutant ubiquitin as indicated. His-pull-down products of ubiquitinated Set8 and WCL were subjected to IB analysis with indicated antibodies.

h. Mass spectrometry analysis to identify the Ser253 phosphorylation event in Set8.

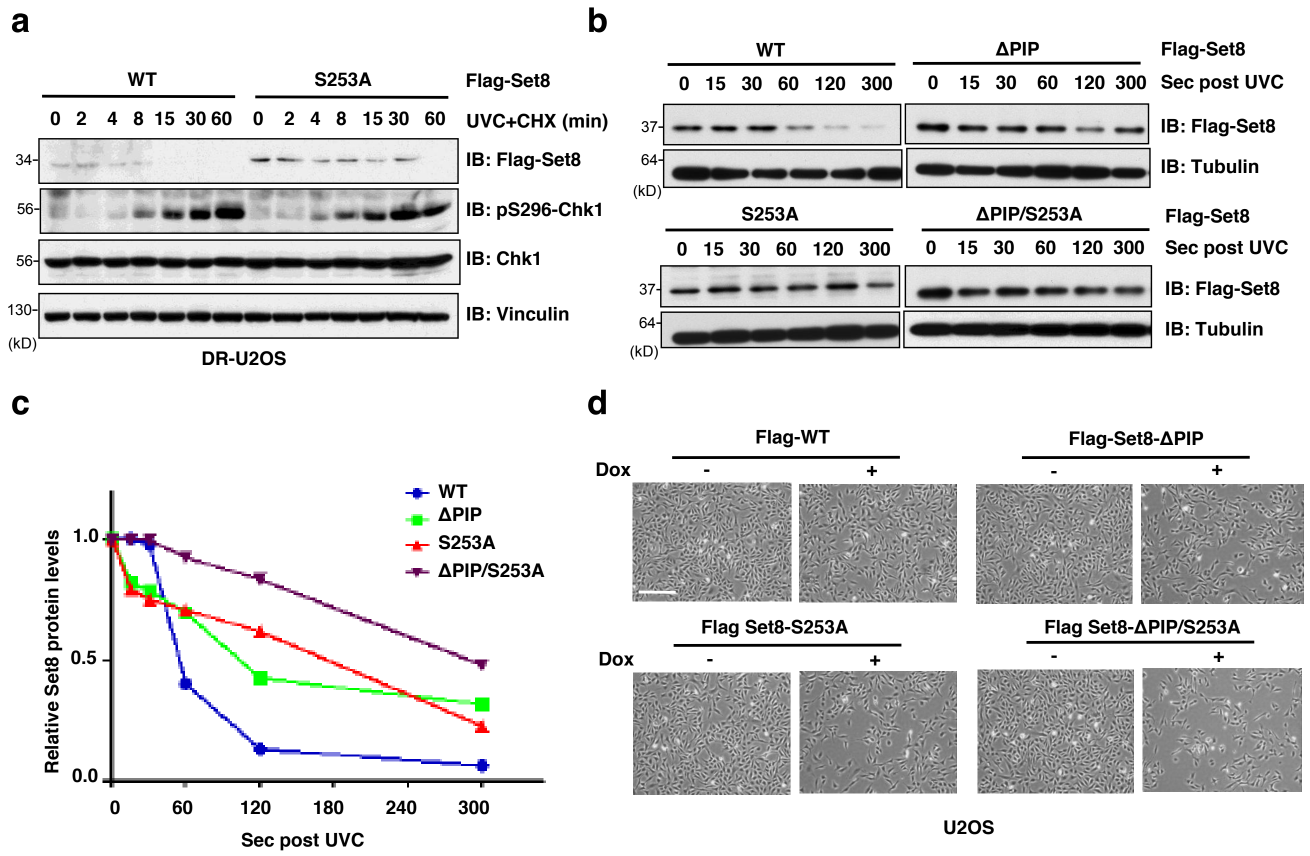

Supplementary Figure 5. Effects of β-TRCP-mediated degradation of Set8 on cell growth.

- **a.** U2OS cells were treated with doxycycline to induce the Set8 WT or S253A expression and then treated with UVC and 20 μg ml^{-1} CHX for different time periods. At the indicated time points, WCL were prepared, and immunoblots were probed with the indicated antibodies.
- **b.** U2OS cells with induced Flag-Set^{WT}, $Set8^{\Delta PIP}$, $Set8^{S253A}$ or $Set8^{\Delta PIP/S253A}$ were treated with UV (50 J m^{-2}), IB analysis was performed to analyze the levels of Flag-Set8 and tubulin.
- **c.** Quantification of the Set8 band intensities in (b). Set8 band intensity was normalized to tubulin.
- **d.** U2OS cells harboring Flag-$Set8^{WT}$, $Set8^{\Delta PIP}$, $Set8^{S253A}$, or $Set8^{\Delta PIP/S253A}$ were cultured in the absence or presence of 0.1 μg ml^{-1} doxycycline (Dox). The images were taken by microscopy to show a representative image of cell proliferation status. Scale bar: 100 μm

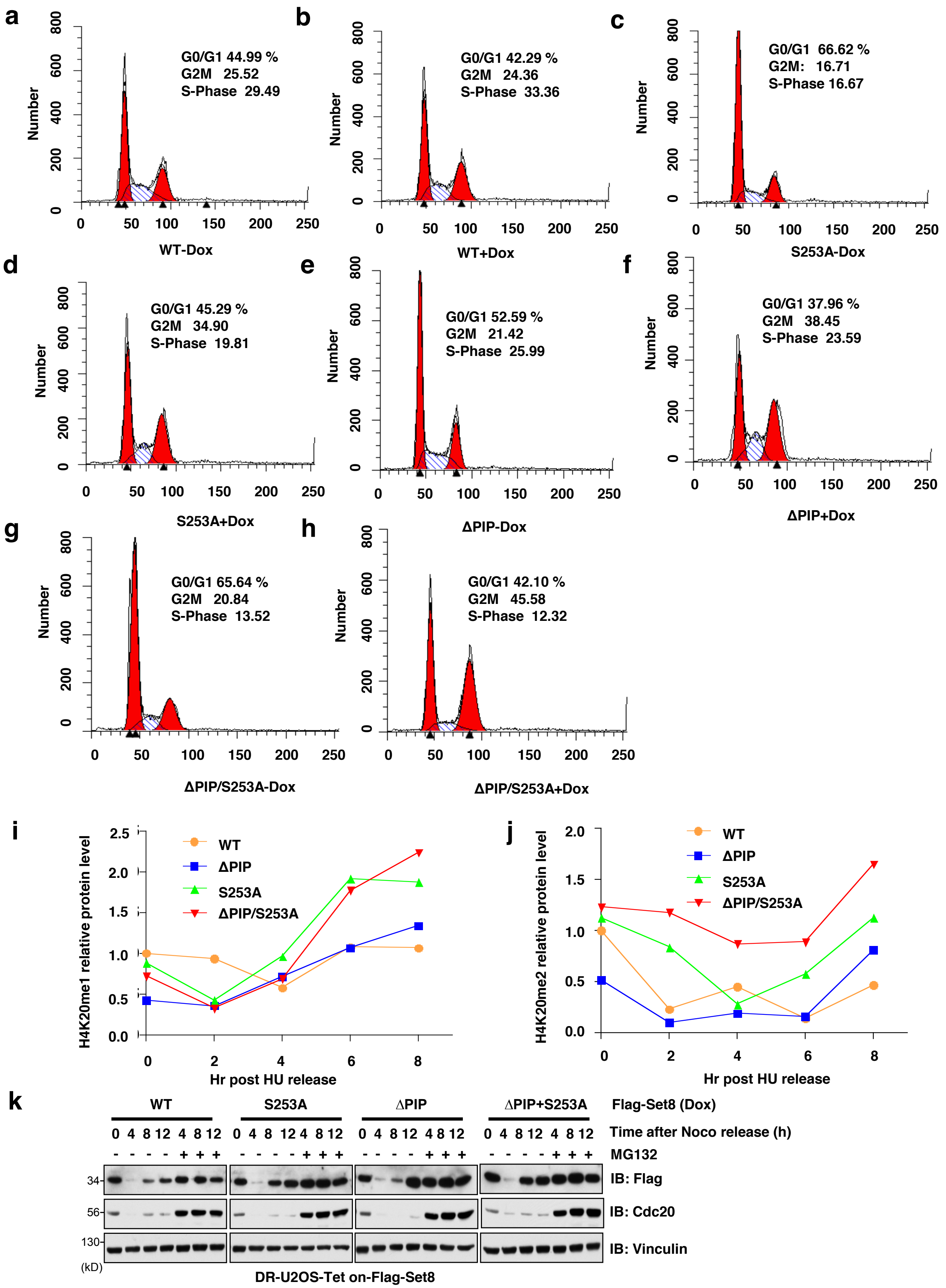
a
G0/G1 44.99 %
G2M 25.52
S-Phase 29.49
WT-Dox
b
G0/G1 42.29 %
G2M 24.36
S-Phase 33.36
WT+Dox
c
G0/G1 66.62 %
G2M: 16.71
S-Phase 16.67
S253A-Dox
d
G0/G1 45.29 %
G2M 34.90
S-Phase 19.81
S253A+Dox
e
G0/G1 52.59 %
G2M 21.42
S-Phase 25.99
ΔPIP-Dox
f
G0/G1 37.96 %
G2M 38.45
S-Phase 23.59
ΔPIP+Dox
g
G0/G1 65.64 %
G2M 20.84
S-Phase 13.52
ΔPIP/S253A-Dox
h
G0/G1 42.10 %
G2M 45.58
S-Phase 12.32
ΔPIP/S253A+Dox
Number
i
H4K20me1 relative protein level
WT
ΔPIP
S253A
ΔPIP/S253A
Hr post HU release
j
H4K20me2 relative protein level
WT
ΔPIP
S253A
ΔPIP/S253A
Hr post HU release
k
WT
S253A
ΔPIP
ΔPIP+S253A
Flag-Set8 (Dox)
Time after Noco release (h)
MG132
IB: Flag
IB: Cdc20
IB: Vinculin
34-
56-
130-
(kD)
DR-U2OS-Tet on-Flag-Set8

Supplementary Figure 6. β-TRCP-mediated degradation of Set8 affects cell cycle.

a-h. U2OS cells harboring Flag-Set8^{WT} (**a**, **b**), Set8^{S253A} (**c**, **d**), Set8$^{\Delta PIP}$ (**e**, **f**), or Set8$^{\Delta PIP/S253A}$ (**g**, **h**) were cultured in the absence or presence of 0.1 μg ml^{-1} doxycycline (Dox). Cell cycle was analyzed by PI staining using FACS.

i-j. Quantification of the H4K20me1 (i) and H4K20me2 (j) bands intensities in Fig. 6b. The band intensity was normalized to Vinculin.

k. U2OS cells harboring inducible Flag-Set8^{WT}, Set8^{S253A}, Set8$^{\Delta PIP}$, or Set8$^{\Delta PIP/S253A}$ were synchronized in M phase with nocodazole for 18 hours. During the last 4 hours in nocodazole, expression of Set8 was induced by doxycycline (Dox). Then, cells with or without MG132 treatment were released from nocodazole in the presence of Dox, and IB analysis was performed to detect the indicated proteins.

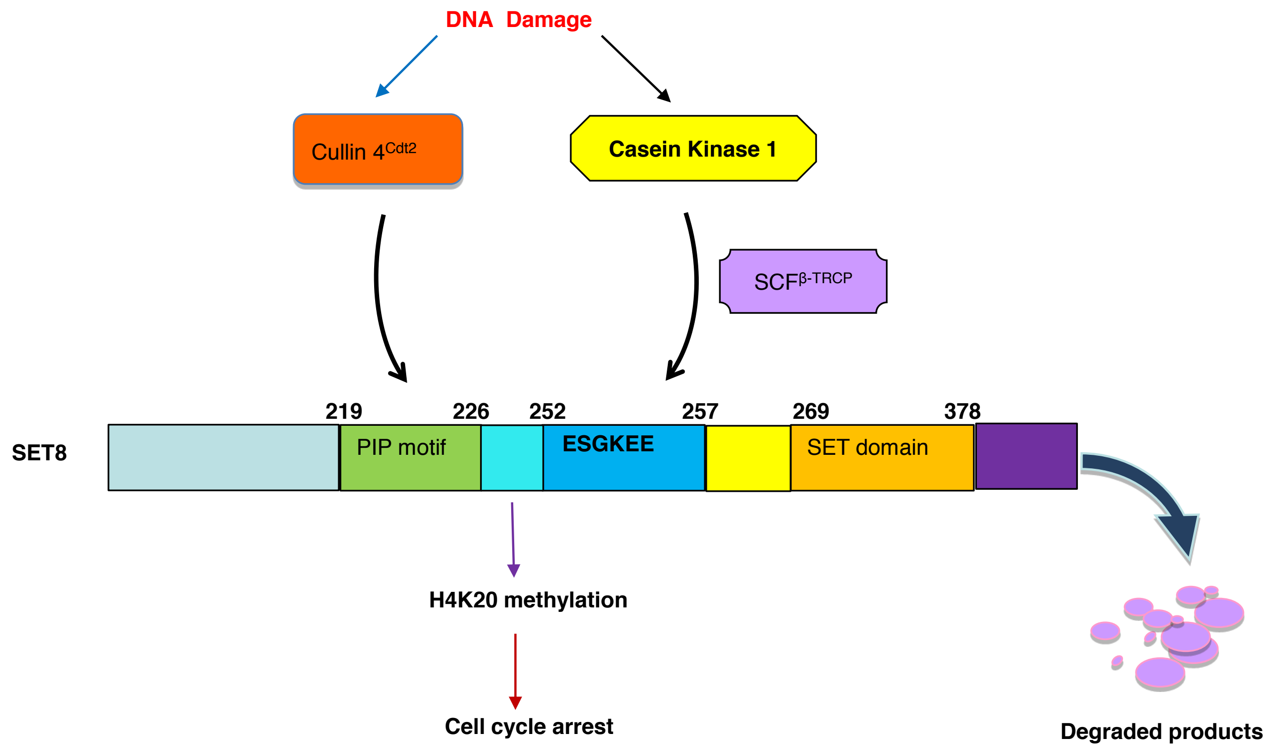

Supplementary Figure 7. A schematic model showing mechanistically how both β-TRCP and Cdt2 regulate Set8 during the cell cycle progression.

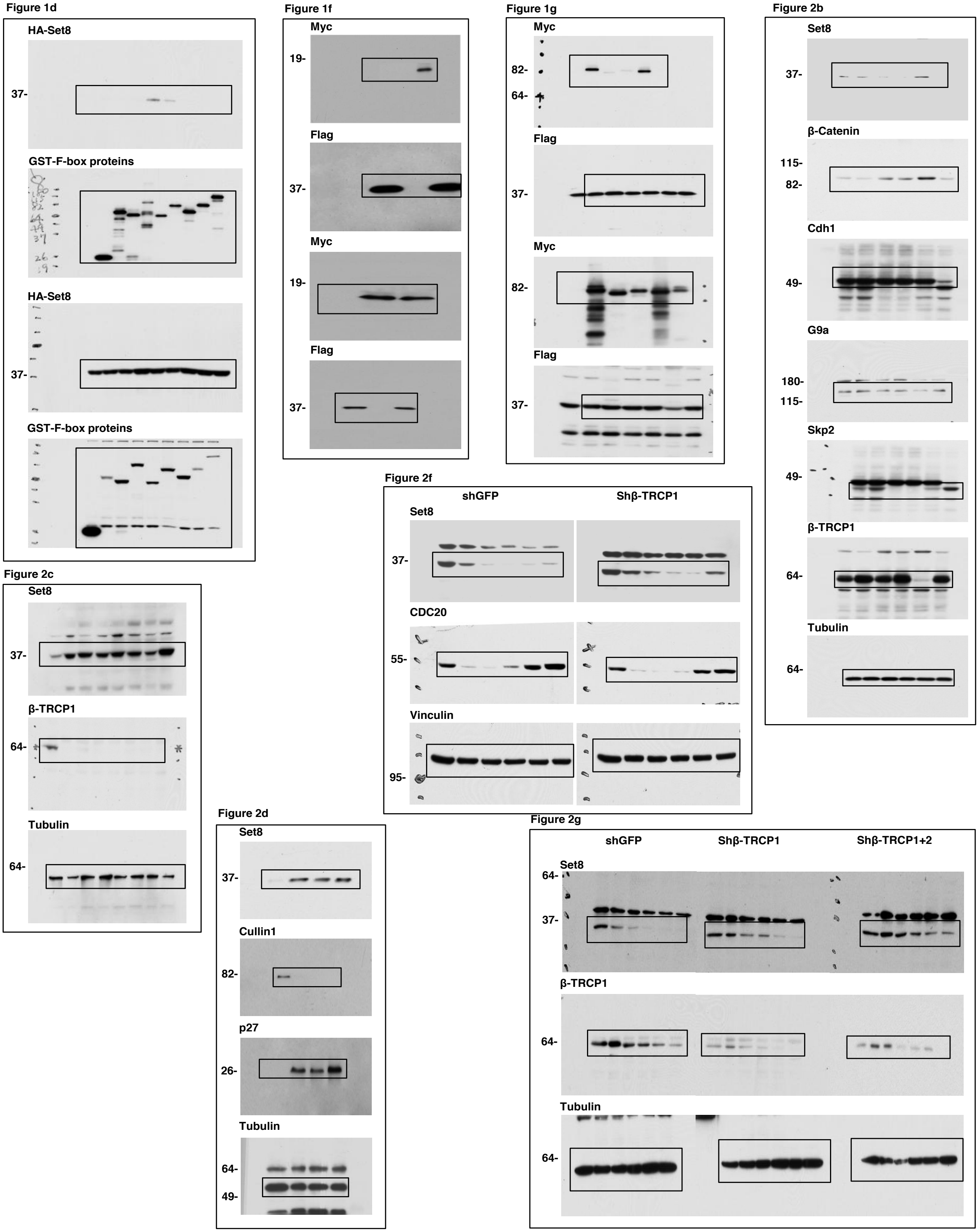

Supplementary Figure 8a. Full images of the most important immunoblots in Figure 1 and 2. Small black boxes show approximate image used for presentation.

Figure 3a

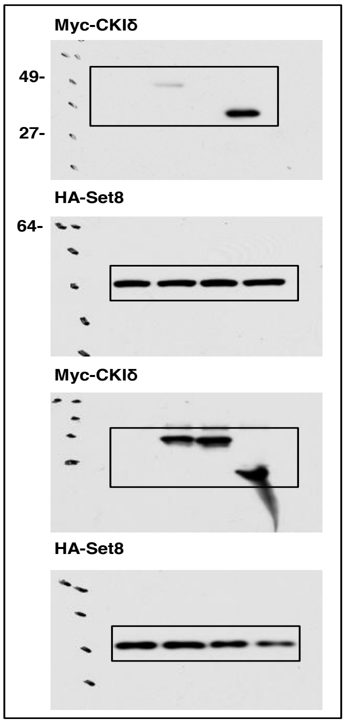

Figure 3b

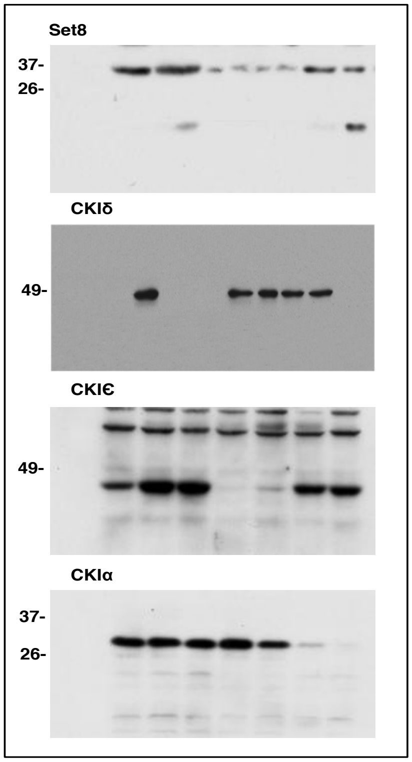

Figure 3c

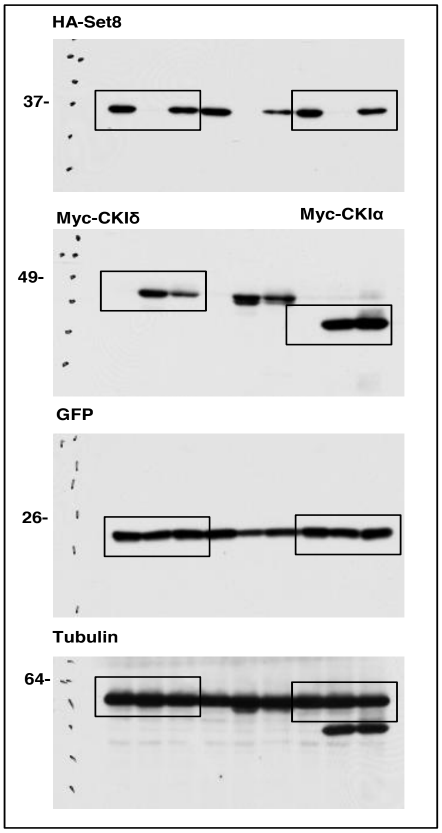

Figure 3d

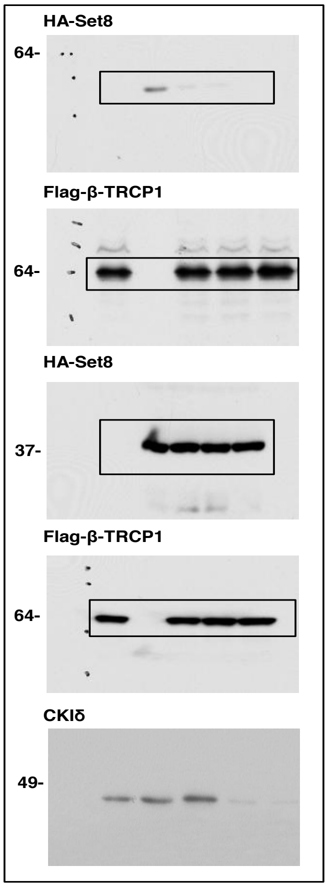

Figure 3f

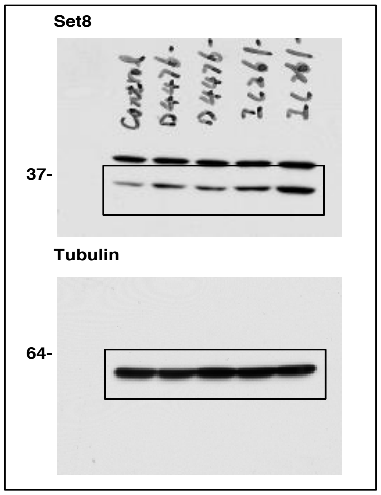

Figure 3g

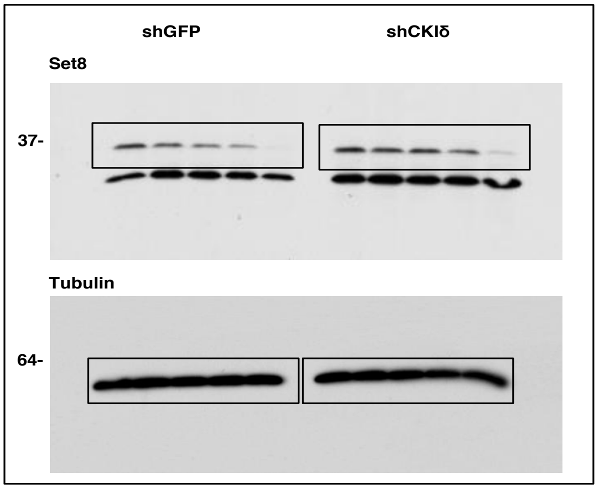

Supplementary Figure 8b. Full images of most important immunoblots in Figure 3. Small black boxes show approximate image used for presentation.

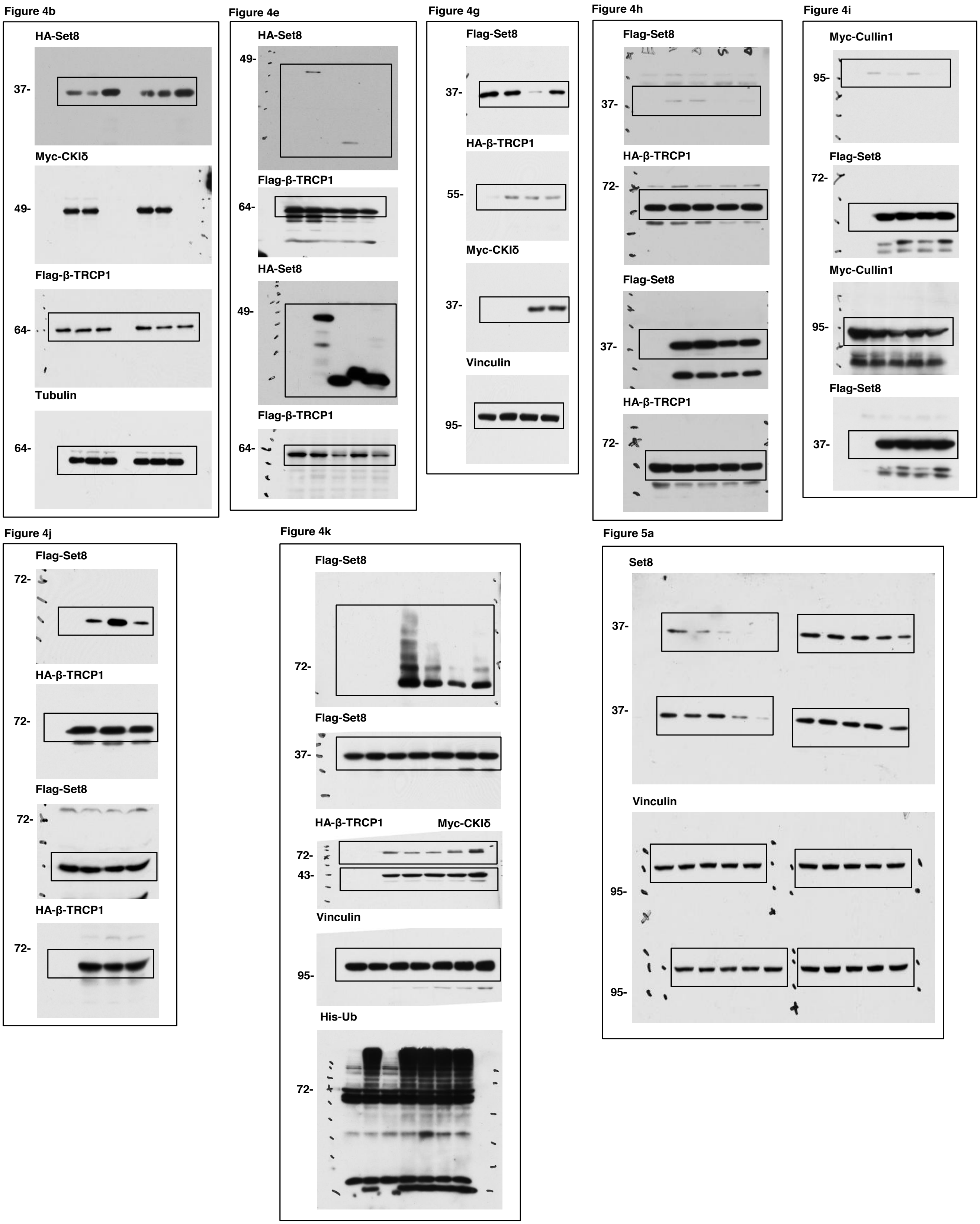

Supplementary Figure 8c. Full images of most important immunoblots in Figure 4 and Figure 5. Small black boxes show approximate image used for presentation.

Figure 5c

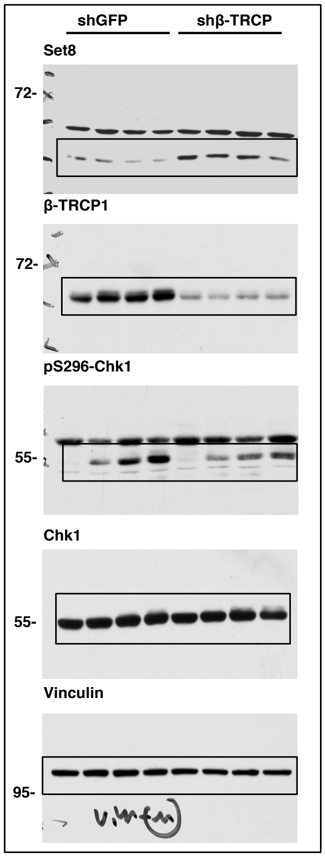

Figure 6b

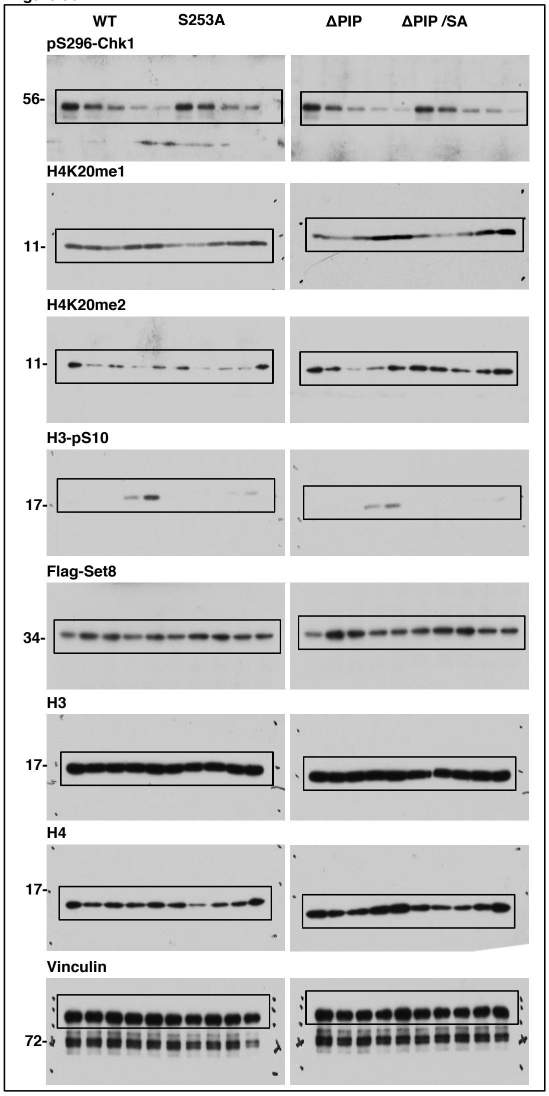

Supplementary Figure 2e

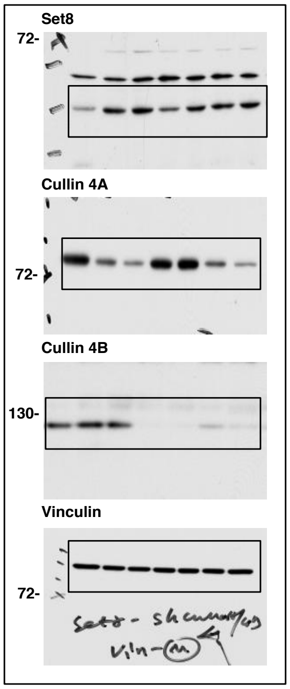

Supplementary Figure 2h

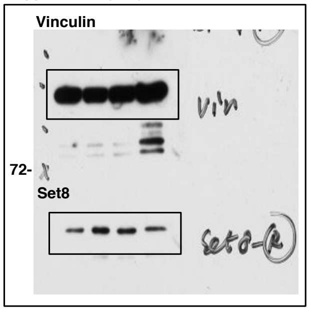

Figure 6c

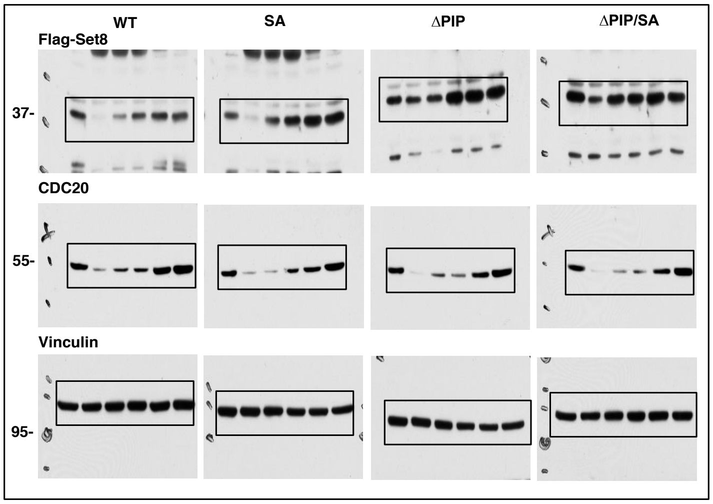

Supplementary Figure 8d. Full images of most important immunoblots in Figure 5, Figure 6 and Supplementary Figure 2. Small black boxes show approximate image used for presentation.
